# Paper-Based Humidity Sensors as Promising Flexible Devices, State of the Art, Part 2: Humidity-Sensor Performances

**DOI:** 10.3390/nano13081381

**Published:** 2023-04-16

**Authors:** Ghenadii Korotcenkov, Nikolay P. Simonenko, Elizaveta P. Simonenko, Victor V. Sysoev, Vladimir Brinzari

**Affiliations:** 1 Department of Physics and Engineering, Moldova State University, MD-2009 Chisinau, Moldova; vbrinzari@mail.ru; 2 Kurnakov Institute of General and Inorganic Chemistry, The Russian Academy of Sciences, 31 Leninsky pr., 119991 Moscow, Russia; n_simonenko@mail.ru (N.P.S.); ep_simonenko@mail.ru (E.P.S.); 3 Department of Physics, Yuri Gagarin State Technical University of Saratov, 77 Polytechnicheskaya str., 410054 Saratov, Russia; vsysoev@sstu.ru

**Keywords:** capacitive, conductometric, impedance, shape deformation, fiber-optic, RFID, polymers, CNTs, graphene, solid state, composites, response, time of response and recovery, stability, challenges, trends

## Abstract

This review article covers all types of paper-based humidity sensor, such as capacitive, resistive, impedance, fiber-optic, mass-sensitive, microwave, and RFID (radio-frequency identification) humidity sensors. The parameters of these sensors and the materials involved in their research and development, such as carbon nanotubes, graphene, semiconductors, and polymers, are comprehensively detailed, with a special focus on the advantages/disadvantages from an application perspective. Numerous technological/design approaches to the optimization of the performances of the sensors are considered, along with some non-conventional approaches. The review ends with a detailed analysis of the current problems encountered in the development of paper-based humidity sensors, supported by some solutions.

## 1. Introduction

The first part of this literature review [1] primarily considered the advantages of the use of paper to develop cheap, flexible humidity sensors and discussed the general approaches that can be employed while fabricating paper-based (PB) humidity sensors. However, these sensors might rely on a number of measuring principles, including capacitive, resistive, and impedance values, as detection signals. They can be fully designed as paper units or involve the application of various humidity-sensitive materials, such as carbon nanotubes, graphene, and many other solid-state and polymer materials, to the paper surface. This diversity affects the requirements of the materials, technologies, and measurement methods applied in sensor design. Therefore, this topic requires special consideration, which the major aim of this part of the review. We start this part of our review with a discussion of paper-based (PB) capacitive sensors, and then analyze resistive and impedance humidity sensors. Fiber-optic, mass-sensitive, and shape-deformation sensors, as well as RFID (radio-frequency identification) sensors and PB-humidity indicators are also considered in this paper. Finally, we explore current problems observed in the research in and development of paper-based humidity sensors and suggest some approaches to solving the issues of concern. The general problems regarding the manufacturing of humidity sensors and their utilization in practice are thoroughly discussed elsewhere [2,3,4,5,6,7,8,9,10,11,12,13,14,15].

## 2. Capacitance-Based Humidity Sensors

A wide variety of paper types have been tested to manufacture capacitive humidity sensors. In particular, Courbat et al. [16] used p_e: smart paper type 2 from Felix Schoeller, Mraovic et al. [17] used Vimax (recycled paper) (70 g/m^2^), M-Liner (cardboard) (230 g/m^2^), and PackPro (food-packaging paper) (50 g/m^2^), and Andersson et al. [18] tested HP advanced photo paper and Canon PT-101 photo paper. Furthermore, Balde et al. [19] employed a coated paper (65 g/m^2^), Wawrzynek et al. [20] fabricated sensors using 300-µm-thick glossy coated paper from Kelly Paper, Ullah et al. [21] suggested using tissue paper for the fabrication of capacitive humidity sensors, and Gaspar et al. [22] fabricated sensors based on Lumi silk (90 g/m^2^) from Stora Enso (Helsinki, Finland).

**Figure 1:**
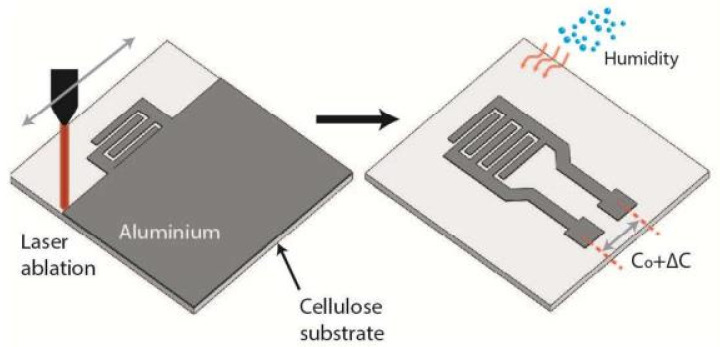
Schematic illustration of the fabrication procedure for capacitive humidity-sensor laser ablated onto MP. Reprinted with permission from [24]. Copyright 2018: ACS.

Although parallel-plate (PP) capacitive sensors generally have a higher sensitivity than IDE-based capacitive sensors [23], these structures are surprisingly reported to be rather sporadic [21]. Much more often, humidity sensors are manufactured using interdigitated electrodes, which may be due to their easier availability in microelectronic laboratories and enterprises. For the manufacture of ID electrodes, various types of printing technology are commonly used. However, Rahimi et al. [24] showed that the laser-ablation method can also be used to form IDE on the paper surface, which can become a simple and scalable alternative to conventional photolithography-based processes and printing technologies. They tested two laser-processing methods for the selective removal of the conductive aluminum film (25 nm) of a metallized paper (MP) substrate, namely direct (DLA) and indirect laser ablation (ILA), for operation at wavelengths of 1.06 µm (Nd-YAG) and 10.6 µm (CO_2_), respectively. Metalized paper (MP) is a commodity in which a thin layer of aluminum offers both a decorative appearance and protective/controlled gas permeation. Rahimi et al. [24] found that the utilization of a Nd:YAG (1.06 µm) fiber laser, which had a greater absorption coefficient for metal (Al) films than the paper substrate, can provide more direct laser ablation (DLA), enabling higher selectivity in the removal of metallized layers, with minimal thermal effects on the porous paper substrate. This process is shown schematically in Figure 1. Unlike the ILA, the DLA did not damage the paper substrate. In addition, this process provided more uniform and straight metal lines than the CO_2_-based laser-ablation process.

In general, the humidity-sensing materials employed in PB sensors might be classified into two types: (i) the paper itself, and (ii) non-paper hydrophilic humidity-sensing materials, such as ceramics, polymers, carbon materials, and their composites. Below, we summarize the development status of PB humidity sensors according to the types of the materials used in their design.

### 2.1. Capacitive Humidity Sensors with Paper as the Sensing Material

Table 1 lists the characteristics of PB humidity sensors with paper as the sensitive material. To characterize capacitive humidity sensors, such parameters as sensory response (C/C_0_) and sensitivity (ΔC/ΔRH) are usually used, which describe the change in capacitance upon interaction with humid air, as well as the response time (τ_res_) and recovery time (τ_rec_), which characterize the kinetics of the sensory response. The response/recovery time is usually defined as the time taken to reach 90% of the final equilibrium value. A parameter such as hysteresis is also used, which characterizes the possible differences between sensor readings when measuring in conditions of increasing or decreasing humidity. The methods for measuring these parameters do not differ from the methods used in the testing of gas sensors and described in many publications, including [2,3,4,5,6,9,10].

**Figure 2:**
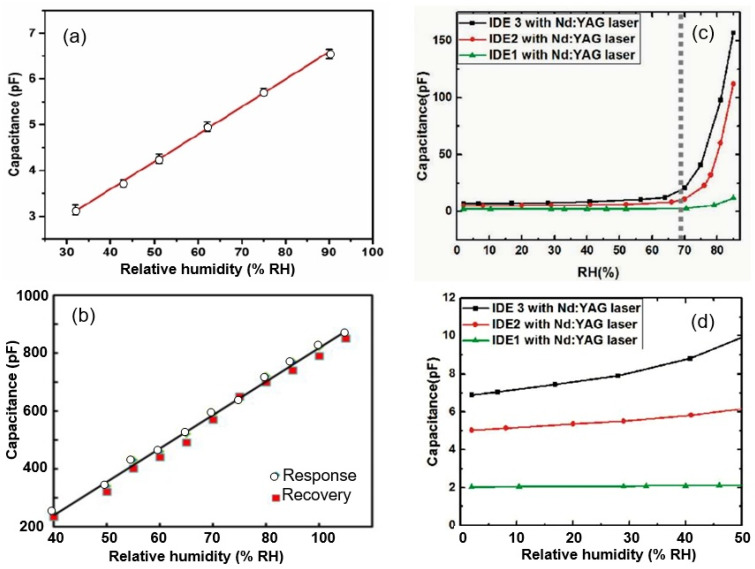
(**a**) Capacitance of the pencil-drawn paper-based sensor with relative humidity. Reprinted with permission from [26]. Copyright 2017: Wiley. (**b**) Response and recovery of parallel-plate capacitive humidity sensor. One plate in the capacitor is composed of a plane of copper tape, while the other plate is composed of an array of meshes. Reprinted from [21]; published 2022 by MDPI as open access. (**c**,**d**) Characteristics of humidity sensors fabricated using Nd:YAG laser ablation, (**c**) capacitance changes with relative humidity levels for the 2–85% RH range, and (**d**) close-up of capacitance changes with relative humidity in the range of 2–50%. By changing the electrode width, the number of electrodes (*n*) covering the effective sensory area of 8 × 8 mm^2^ was changed from *n* = 2 to *n* = 20 and *n* = 40. With the increase in the number of electrodes, the distance between them naturally decreased. These sensors are designated as IDE1, IDE2, and IDE3 respectively. It can be seen that a decrease in the distance between the electrodes leads to a significant increase in the sensory response to changes in humidity. Reprinted with permission from [24]. Copyright 2018: ACS.

The typical characteristics of humidity sensors employing paper-based sensing elements are shown in Figure 2. It is shown that the sensors allow the measurement of air humidity in a wide range. Furthermore, the sensors can have both linear and non-linear characteristics (see Figure 2). Analyzing the obtained results, it can be assumed that the differences are due to differences in the porosity of the paper used and geometry of the formed electrodes. Large pore sizes do not favor capillary condensation, which is usually responsible for strong capacitance growth at high humidity levels. Regarding the geometry of the contacts, Figure 2c,d show that the capacitance variations versus the relative humidity levels increasingly approach a linear form as the distance between the electrodes increases. Interestingly, when comparing the parameters of sensors made using recycled paper, cardboard, and food-packaging paper, Mraovic et al. [17] found that sensors’ parameters, such as their response and the range of measured RH, are weakly dependent on the type of paper used. The types of paper used appeared to differ little in terms of their parameters.

**Figure 3:**
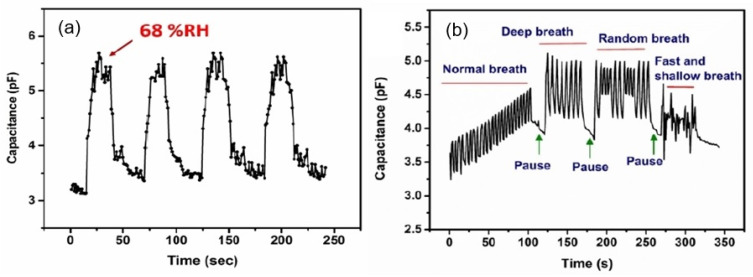
(**a**) Dynamic response of pencil-drawn PB sensor at 68% RH. (**b**) The breathing pattern of a subject under testing, measured in terms of capacitance as a function of time with normal breathing, deep breathing, pause in breathing, and random breathing. By interfacing the sensor with the microcontroller, the capacitance data were acquired and transferred to a smartphone through Bluetooth communication. Reprinted with permission from [26]. Copyright 2017: Wiley.

It was also found that the capacitive response is reversible (Figure 3a), and the sensitivity, rate of response, and flexibility of the sensors are sufficient for a variety of applications [26,28]. For example, such sensors can be utilized in measuring rates and patterns of breathing (see Figure 3b). Most humidity sensors are fairly stable for at least 7 days [21]. This may be acceptable for cheap sensors, which are not intended for long-term operation, especially in aggressive environments.

However, the analysis of published results indicates that despite the significant progress in paper-based humidity sensors, most sensors still exhibit poor repeatability, high drift (increase in base value (C_0_) above 10%), low sensitivity (C/C_0_ is typically below 200%), and slow response/recovery (from 4–16 s to several minutes) [17,22]. In addition, some of the technology routes (protocols) used in the course of producing the humidity sensors, such as tape pasting, vacuum filtration, and hand drawing, are not sufficiently scalable and reliable for mass production. Meanwhile, recent studies revealed that many of the problems associated with paper-based humidity sensors can be successfully resolved. For example, Zhang et al. [27], developed capacitive humidity sensors with a maximum change of 1480% (C/C_0_) at 1 kHz, and a maximum change of 411.4% at 10 kHz, using robust and precise screen- and gravure-printing technologies. The sensors were highly repeatable, exhibiting a standard deviation in sensitivity of 1.05% for three continuous runs, stable, and insensitive to mild temperature changes (15–30 °C). It is worth noting their fast response, of ca. 0.8 s, and recovery, of ca. 0.78 s. To achieve these results, Zhang et al. [27] subjected the copy paper to a treatment in 200 mM of hydrochloric acid (Sigma-Aldrich, St. Louis, MO, USA) to remove the calcium-carbonate filler. Next, the as-prepared porous paper was pressed and dried for 180 s at 115 °C. Compared with the conventional paper, the paper prepared in this manner had a larger specific surface area, which provided, according to Zhang et al. [27], remarkable sensor performance. The sensors, fabricated by Rahimi et al. [18], also showed a high degree of repeatability, with a capacitance variability of less than 4%.

However, the information given in Table 1, especially regarding the response/recovery times, should be treated with caution, because these measurements are often carried out in large measuring chambers. This means that these data may receive a substantial contribution from the dynamics of atmospheric changes in the chamber. Furthermore, high sensitivity, measured as ∆C/% RH (pF/% RH), does not always take into account the area of the capacitive sensor, which masks the real value. Frequently, to achieve high sensitivity, it is sufficient to simply enhance the sensor area.

### 2.2. Humidity Sensors with Solid-State Sensing Elements

Some solid-state materials, such as carbon nanotubes (CNTs), graphene oxide (GO), porous silicon (PSi), and alumina oxide (AO), have been applied to produce capacitive paper-based humidity sensors. The parameters of these sensors are listed in Table 2.

As can be seen from the results presented in Table 2, sensors designed with GO and CNTs, except for the sensors developed by Song et al. [33], are not highly sensitive to water vapor [30,32], which coincides with the results of studies performed on polymers and solid substrates [10,11]. Moreover, their parameters are noticeably inferior to those of Al_2_O_3_-based detectors [19] and PSi [35]. However, this is quite understandable because the nanoporous structures of Al_2_O_3_ and PSi encourage the capillary condensation of water vapor, which has a significant effect on the material capacitance (or dielectric permittivity) compared to the adsorption of H_2_O molecules on the surfaces of GO and CNTs only. Regarding the sensors developed by Song et al. [33], high sensitivity was achieved by optimizing the IDE structure and choosing the correct paper substrate.

**Figure 4:**
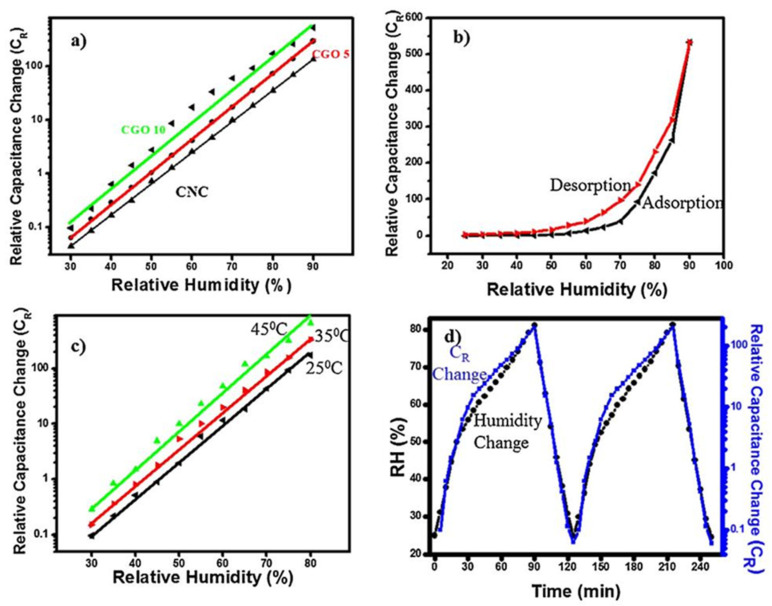
(**a**) Relative capacitance change (CR) with relative humidity change for pristine CNCs, CGO5, and CGO10 composite. (**b**) Humidity-hysteresis curve of the sensor for CGO10 composite. (**c**) Relative capacitance change with humidity at different temperatures. (**d**) Relative humidity change (% RH) and relative capacitance change with time. Reprinted with permission from [34]. Copyright 2016: Elsevier.

Recently, a remarkably high sensitivity to humidity was reported for sensors based on GO/CNC composites; the data are displayed in Figure 4 [34]. In this case, the inclusion of cellulose nanocrystals (CNCs) in the composites, which had pronounced hydrophilic properties, produced highly adsorbent H_2_O molecules. In addition, CNCs have large specific surface areas and high mechanical strength, which are important for sensing units. Cellulose nanocrystals are mainly derived from naturally occurring cellulose fibers by removing amorphous segments through strong-acid hydrolysis. In the study, to form a GO/CNC composite, the GO suspension was mixed with the CNC composite at the desired ratio, of 5 wt.% and 10 wt.% of CNC, followed by homogenization. Further, an electrode with an interdigital transducer (IDT) pattern was deposited on a PET substrate using a conventional lift-off process. Next, the CNC/GO solution was poured dropwise onto the patterned electrode and dried in an oven at 60 °C. It should be noted that no paper substrate was used in the manufacturing of this sensor, which distinguishes it from those considered above.

**Figure 5:**
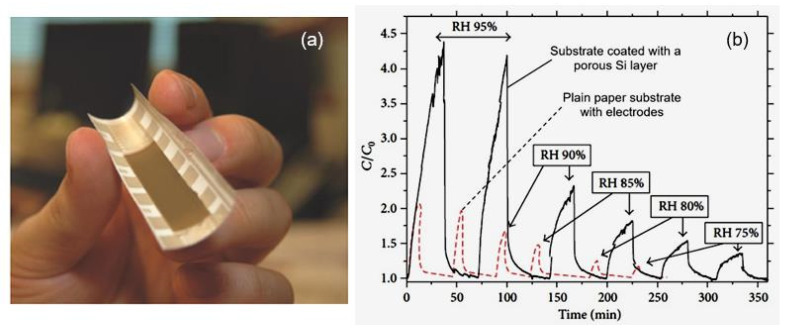
(**a**) A photograph showing a spray-coated PSi layer on flexographically printed silver electrodes. (**b**) Relative capacitive response of paper-based sensor to changes in RH concentration, showing that when the thickness of the sensing layer is suitably thick, the signal from the sensing layer is clearly stronger than the signal from the substrate. Reprinted from [35]. Published 2015 by Hindawi as open access.

Previously, Jalkanen et al. [35] showed that porous Si nanoparticles can be suitable materials with which to design humidity sensors because (i) they have a large active surface area and (ii) their surface chemistry can be modified to accommodate a wide range of environmental and biochemical sensing applications. The sensing elements, consisting of printed ID Ag electrodes and a PSi layer, were fabricated on a coated paper substrate via a two-step process. An image of the PSi-based humidity sensor is given in Figure 5a. The PSi particles were dispersed in a suspension of toluene. A wide particle-size distribution, ca. 100–2000 nm, was found to improve the packing and adhesion of the PSi film over the paper substrate. Various ID electrodes patterned with diverse gaps, measuring 100–500 µm, were supplied to the substrate by flexography and inkjet printing. Jalkanen et al. [35,36] believe that the technology routes applied to fabricate sensors are easily scalable for mass production. The test results are shown in Figure 5a. It can be seen that the maximum sensory response was observed in the range of relative humidity of 70–95% and increased in line linearly with the increase in the water-vapor concentration. In this range of relative humidity, it was possible to detect changes in H_2_O content of less than 5% RH. However, it was also clear that the sensors had a limited sensitivity range because the sensory response was rather low, in the RH < 70% range. In addition, these sensors had long response and recovery times. It was also found that the sensors had significant hysteresis characteristics.

**Figure 6:**
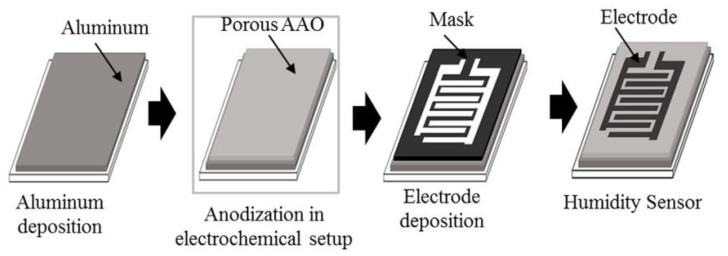
Process of fabrication of AAO-based humidity sensor. Reprinted with permission from [19]. Copyright 2015: Elsevier.

Balde et al. [19] used the opposite approach. Instead of applying a sensitive layer to the surface of the paper substrate, they formed a layer of porous aluminum directly on the paper. For this purpose, anodic oxidation of the aluminum layer deposited on the surface of coated paper (65 g/m^2^) was used. The ID-electrode capacitor was fabricated by an evaporation of 300-nm-thick aluminum onto the top of a thin AAO film through a Pt mask (see Figure 6).

**Figure 7:**
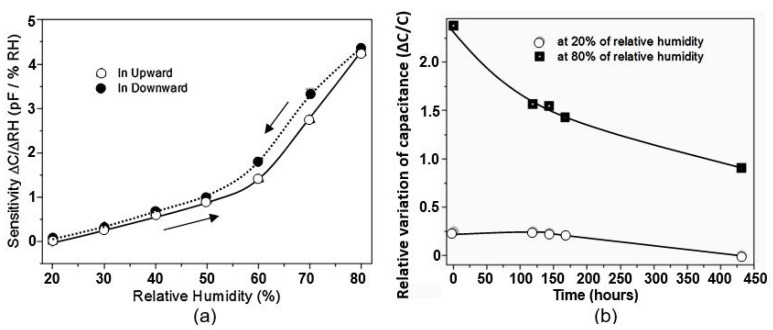
(**a**) Sensitivity-hysteresis characteristics of AAO-based PB sensor with 100 V of applied voltage at 1 kHz. (**b**) Relative capacitance variation of sensor with AAO formed at 100 V measured at 1 kHz five times over a period of 2 weeks Reprinted with permission from [19]. Copyright 2015: Elsevier.

In general, humidity sensors based on AAO are highly sensitive, as shown in Figure 7a. However, at the same time, it was found that these sensors have large hysteresis due to the capillary absorption of water into the pores. Capillary-condensed water has a much more highly developed hydrogen bond than that which appears when water molecules adsorb on a flat surface. In addition, there temporary changes occur in sensor readings during prolonged exposure to humid atmospheres (Figure 7b). Due to the specific structure of the sensitive layer, these sensors are also subject to a greater influence from the mechanical loads that occur when the substrates are bent.

It is important to note here that the observed sensory response and hysteresis in paper-based sensors using an additional coating with a humidity-sensitive material should be considered as the combined response of the paper substrate with pronounced hydrophilic properties and the sensitive material, which inhibits the adsorption/desorption of H_2_O molecules on the substrate [32]. In these structures, the passivation of the paper substrate prior to the deposition of additional humidity-sensitive materials may be a rational solution to improve the sensory-response kinetics. This conclusion is in good agreement with the results of studies conducted by Kafy et al. [34], who found that GO/CNC-based sensors fabricated on a PET polymer substrate performed faster than paper-based devices (see Table 2).

## 3. Conductometric (Resistive) Humidity Sensors

Conductometric paper-based humidity sensors are the most frequently studied sensor types. They are distinguished by their ease of manufacture and operation, as well as high sensitivity. Currently, many scientific groups are working in this area. These groups have shown that all types of humidity-sensitive material can be used to develop conductometric paper-based sensors, from paper- and carbon-based materials to solid-state materials, polymers, and various composites.

### 3.1. Resistive Humidity Sensors with Paper as the Sensing Material

The parameters of humidity sensors which employ a paper as a detector element are presented in Table 3. It can be observed that resistive humidity sensors are characterized using the same parameters as capacitive sensors. This is sensory response is calculated as the ratio of resistance (R_0_/R) or current (I/I_0_) to changes in humidity, the sensitivity calculated as the ratio of changes in resistance to changes in humidity (ΔR/ΔRH), and the response/recovery time determined as mentioned earlier in Section 2.1.

**Figure 8:**
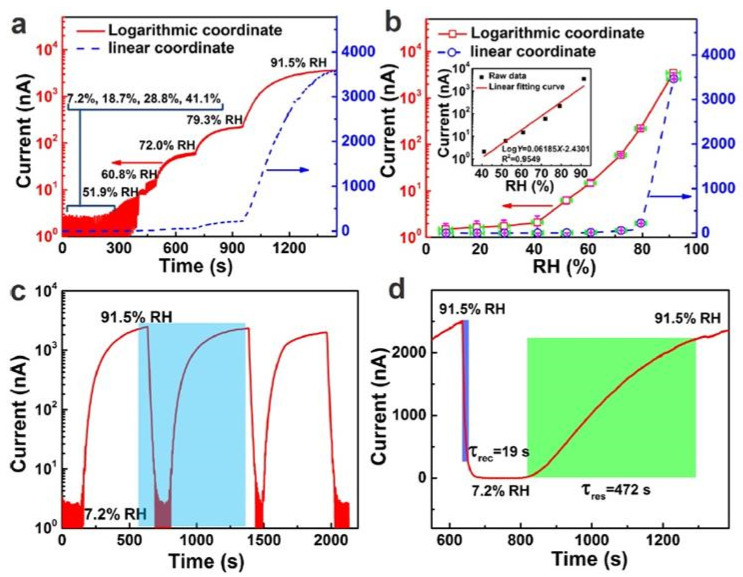
Humidity-sensing properties of the humidity sensor. (**a**) Dynamic-response-characteristic curves of the humidity sensor at different RH. (**b**) Current versus RH curves; the inset shows the linear fitting curve of current versus RH. (**c**) Response and recovery curves for three cycles. (**d**) Amplified response and recovery curve in the linear coordinate system. Reprinted with permission from [37]. Copyright 2019: ACS.

The research data presented in Table 3 show that the conductivity of paper is very sensitive to H_2_O vapors and can vary over a wide range, which makes it possible to develop highly sensitive conductometric (resistive) humidity sensors. However, the conductivity of paper normally appears to be very low, which makes the resistance of the corresponding sensors excessively high for ordinary electronic readout circuits. The conductivity begins to noticeably increase only at humidity levels above 40–50% RH [37,38]. This naturally limits the range of possible applications for these sensors. Of course, this threshold of RH is not universal for all types of paper. For example, Guder et al. [40] compared the parameters of sensors based on three types of paper: (1) Whatman 1 Chr, (2) Whatman 3MM Chr, and (3) copy paper. Both the Whatman 1 Chr and the Whatman 3MM Chr were fabricated from pure cotton-cellulose fibers, with base weights of 87 g·m^−2^ and 185 g·m^−2^, respectively. Standard carbon paper has a density of 80 g·m^−2^. Gooder et al. [40] found that none of the sensors employing these types of paper generated a readable current upon exposure to H_2_O vapors below 20% RH. The Whatman 1 Chr had the lowest sensitivity and did not give a noticeable signal below 65% RH. The Whatman 3MM Chr was significantly more sensitive than the Whatman 1 Chr and detected the humidity at RH > 30%. Gooder et al. [40] believe that the observed differences in sensitivity between the sensors made with Whatman 3MM Chr and Whatman 1 Chr materials may have been related to their density. The larger area of cellulose fibers in the Whatman 3MM Chr yielded more electrically conductive paths at a given level of relative humidity, and thus increased the sensor sensitivity. The copy paper exhibited the highest sensitivity. However, the mechanism underlying these differences is currently unknown. Nevertheless, the high sensitivity of the copy paper compared to the Whatman 3MM Chr was achieved at the cost of a 10-fold increase in sensor-power consumption. Therefore, Gooder et al. [40] concluded that it is preferable to utilize Whatman 3MM Chr paper for these sensor tasks. However, despite these recommendations, copy paper is widely considered in the research on and development of humidity sensors [37,41,44]. Some of the characteristics of the sensors developed by Duan et al. [37] are drawn in Figure 8.

According to Duane et al. [37], the conductivity of printed paper increases dramatically at RH > 40%. In the relative humidity range of 41–91.5%, the sensory response is greater than 10^3^, making these sensors suitable for the accurate detection of H_2_O vapors in a given environment. As shown in Figure 8d, the estimated response/recovery times for the sensor were found to be ca. 472 s and 19 s, respectively. In comparison, the sensors reported later, in [44], had response and recovery times of about 240 and 30 s, respectively. This persistently slow response seems to be unfavorable for some specific applications, in which fast responses are required. However, an experiment performed by Duanet al. [44] ensured that a sensor quickly responded to breathing, with switching times at 1–5-s intervals due to its high sensitivity.

**Figure 9:**
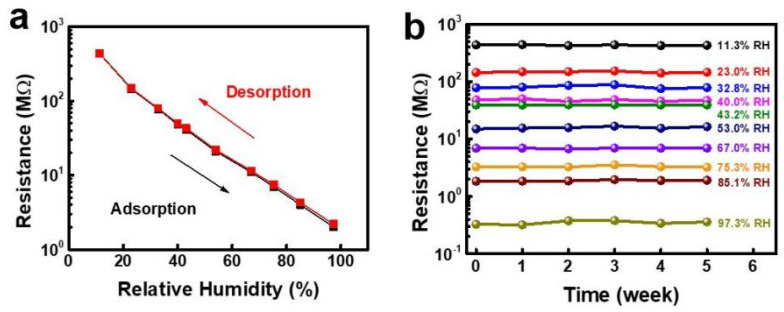
(**a**) The resistance response of CKF in continuous adsorption and desorption measured in the 11.3~97.3 RH% range. (**b**) The resistance response of CKF under different humidity conditions over 5 weeks. Reprinted with permission from [47]. Copyright 2020: ACS.

To expand the sensitivity range of PB humidity sensors, Wang et al. [47] suggested using a transparent and flexible cellulose/KOH composite ionic film (CKF). A cellulose/KOH composite hydrogel film was prepared by extracting a cellulose/BzMe_3_NOH hydrogel film in 4 wt% KOH solution for over 6 h. To prepare the cellulose/BzMe_3_NOH hydrogel film, the cellulose sample was dispersed in an aqueous BzMe_3_NOH solution at room temperature following centrifugation. The as-prepared cellulose/KOH composite hydrogel film was air-dried at room temperature overnight. The cellulose substrate was fabricated through a water-evaporation-induced dense-packing strategy. As demonstrated in Figure 9, the conductive CKF showed a fast and reversible real-time response to relative humidity (RH) in the range of 11.3–97.3% RH. The conductivity changed over 200 times and the response/recovery times were 6.0/10.8 s, which were faster than most of the other times reported in the literature. The hysteresis value was about 0.57%, which was also significantly less than the values given in the literature. In addition, CKF is insensitive to both temperature (10–0 °C) and pressure (0–120 kPa), indicating its high selectivity for humidity sensors. Furthermore, CKF-based sensors also exhibit high stability (see Figure 9b).

**Figure 10:**
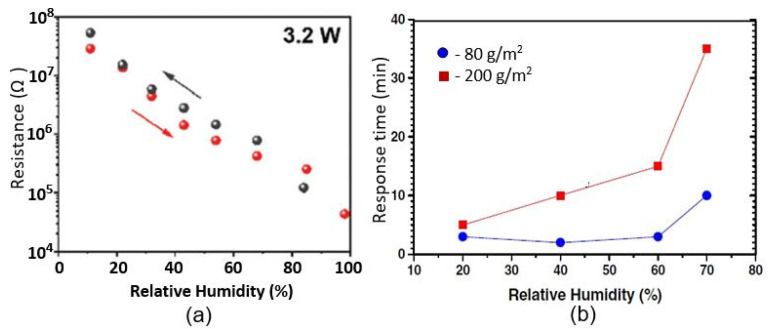
(**a**) Resistance and hysteresis versus relative humidity of TEMPO-oxidized cellulose-based sensors with electrodes formed using laser-irradiation powers of 3.2 W. All performance tests were conducted at room temperature. Reprinted with permission from [45]. Copyright 2022: RSC; (**b**) Comparison of the response times between the devices on the substrate of printing paper (80 grm^−2^) and photographic-quality glossy paper (200 grm^−2^) at RH levels of 20–70%. Reprinted from [41]. Published 2016 by Elsevier as open access.

**Figure 11:**
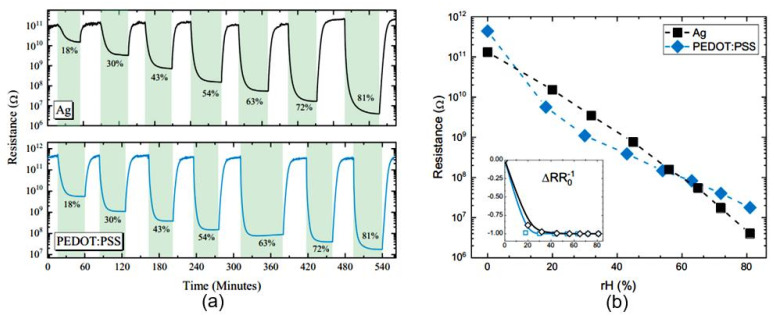
(**a**) Resistance variations of PB humidity sensors with Ag or PEDOT:PSS contacts for different humidity levels. (**b**) The resistance as a function of RH% on logarithmic Y axis. The response can be described with an exponential decay function. Inset plot is the variation with respect to resistance under dry air. Reprinted with permission from [43]. Copyright 2020: Elsevier.

The PB sensor devices based on TEMPO-oxidized cellulose developed by Zhu et al. [45] demonstrated the ability to operate at lower RH values (see Figure 10a). The cellulose was obtained using the TEMPO (2,2,6,6-tetramethylpiperidine-1-oxyl radical)-mediated oxidation of softwood pulp fibers [48] followed by preparing a suspension in water under gentle stirring. The suspension was then filtered, transferred to a glass plate, and dried in an oven to obtain TEMPO-oxidized cellulose paper with a COONa-group content of about 1.8 mmol·g^−1^. However, these sensors also had slow responses. The response and recovery times were about 60 s and 500 s, respectively. Notably, relatively long recovery times are a common problem for sensors employing paper as the humidity-detecting element. This is due to the hydrophilic nature of cellulose, which takes longer to desorb water molecules. Moreover, regularity is frequently observed regarding the effect of paper density: the greater the density of the paper, the slower the sensory response (see Figure 10b). However, this conclusion does not seem to apply to glossy photographic paper. Despite the rather high density of paper, which reaches 135–180 g/m^2^, the sensors using it as a substrate are fast [43]. These sensors have also demonstrated high sensitivity to water vapors (see Table 3 and Figure 11). All photographic papers contain a photosensitive emulsion on their surfaces, consisting of silver halide salts suspended in a colloidal material, such as gelatin, resin, or polyester, which is applied to the paper [49]. However, tests of the reliability upon bending pf sensors made on glossy photographic paper showed that their surfaces are damaged even with fairly slight bending of 9% of the calculated length. Moreover, the fabrication of devices utilizing the PEDOT:PSS humidity-sensing layer failed on these substrates after long-term bending tests. Furthermore, Ag-nanoparticle-derived ink withstood 1000 bending cycles with a low resistance drift.

**Figure 12:**
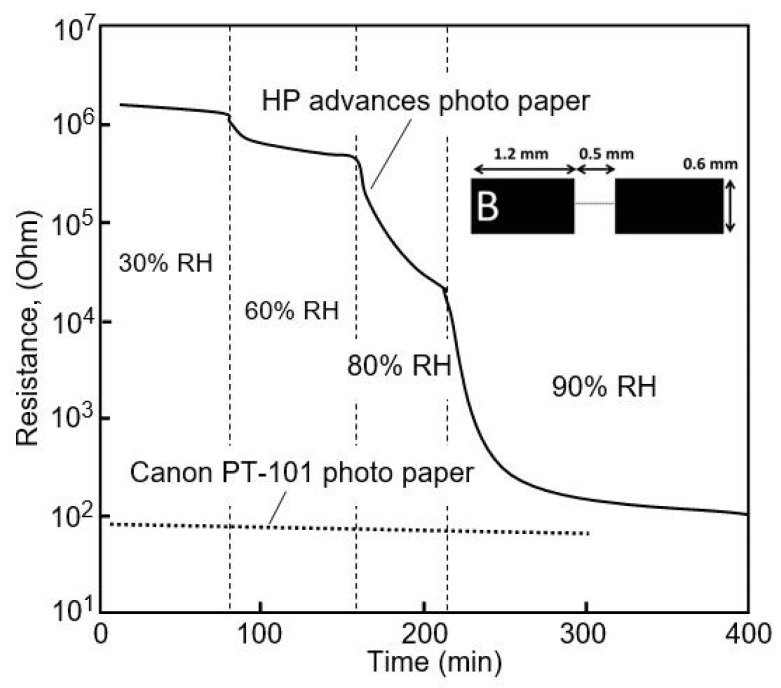
Graph showing the change in resistance over time when sensors of Type B printed on HP Advanced Photo Paper are exposed to 30%, 60%, 80%, and 90% relative humidity at room temperature. It can be seen that the resistance of the sensor based on Canon PT-101 photo paper remains practically unchanged under similar conditions. Data extracted from [18].

Andersson et al. [18] also compared two types of paper, HP advanced photo paper and Canon PT-101 Photo Paper, for application in sensors. Using FTIR, it was determined that the main content of the topcoat was a mixture of SiO_2_ and AlO(OH) in the HP paper and AlO(OH) in the Canon paper. Additionally, no differences in porosity were observed through the Hg-porosimetry measurements. Moreover, the HP paper was found to have significantly higher resistance in a dry environment (see Figure 12), and it was found to be very sensitive to humidity at RH >60%. In addition, the resistivity of the Canon paper changed slightly upon interaction with moisture. After analyzing the results of additional studies, Andersson et al. [18] concluded that the most likely reason for these differences in humidity-detection behavior is the difference in coating chemistry between HP paper and Canon paper. It was found that the sensors printed on Canon paper, although less sensitive to moisture, behaved very similarly to the sensors printed on HP paper following immersion in NaCl solution. This indicates that the resistivity of paper and, hence, its sensitivity to humidity, is determined not only by the properties of the paper itself but also by the chemical composition of its coating. For example, it was observed that coatings containing silicon dioxide, in the case of HP paper, contribute to better sensory performance compared to coatings containing only alumina (Canon paper) [50].

**Figure 13:**
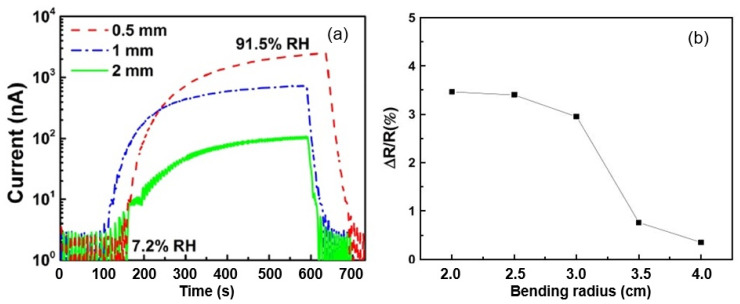
(**a**) Response curves of three humidity sensors with different electrode distances, 0.5, 1, and 2 mm. Reprinted with permission from [37]. Copyright 2019: ACS. (**b**) Percentage change in resistive values of the sensor under different bending conditions (R varied from 4 cm to 2 cm). Reprinted from [46]. Published 2023 by IOP as open access.

It was also been found [37] that the distances between electrodes play an important role in the responses of paper-based resistive humidity sensors. Duan et al. [37] tested sensors with three different electrode spacings in a range of 0.5–2 mm range (see Figure 13). They found that the sensor response (R/R_0_) of the humidity sensor gradually reduced as the distance between the electrodes increased. This effect was also observed by Zhao et al. [51]. According to Zhang et al. [52], the adsorption of H_2_O molecules on paper surfaces creates a discontinuous layer when the gap between two electrodes is excessively large, breaking the percolation conduction channel even at high relative humidity values, which contributes to reductions in sensory response.

With regard to the bendability of resistive humidity sensors on paper, tests carried by Duan et al. [37] suggested that the real-time sensor current has no obvious changes during the bending process. After changing the number of bends from 0 to 200, at a bending angle of 60°, the response and recovery times, as well the responses of the sensors, changed little. This means that paper-based sensors have suitable anti-mechanical flexibility. The wiry geometry of cellulose fibers is highly resistant to stress, and the deformation process causes little or no lateral cracking due to the small diameter of the fibers [53]. Thus, bending does not limit the performances of resistive-type PB humidity sensors. Mansouri et al. [46] also found that bending does not greatly affect the performances of PB sensors. Figure 13 shows the percentage change in the resistance of the sensor when it was bent at 40% RH. The resistance of the sensor increased as the bend radius decreased. It was observed that the sensor resistance changed by 3.46% when the substrate-bending radius altered from R = 4 cm to R = 2 cm. The sensor resistance changed to a much greater extent, i.e., >5%, when the bending radius was less than 2 cm.

### 3.2. Resistive Humidity Sensors with Carbon-Based Sensing Materials

In the manufacturing of paper-based humidity sensors using carbon-based materials as detecting elements, carbon nanotubes (CNTs or MWCNTs), graphene, graphene oxide (GO), reduced graphene (rG), and composites, including carbon and cellulose, have been applied. The parameters of these sensors are listed in Table 4.

**Figure 14:**
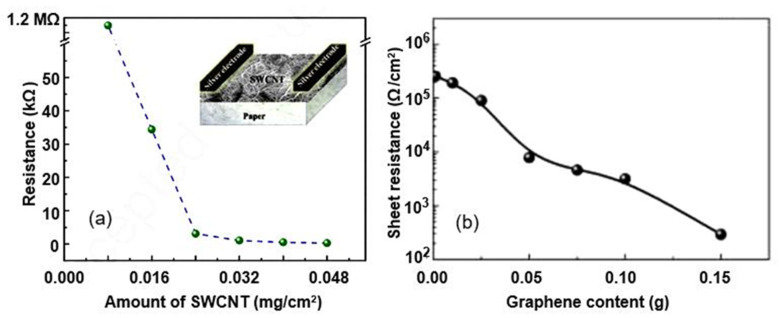
(**a**) Sheet resistance of filter paper with different amounts of SWCNT. The process of fabrication of SWCNT on paper substrate combines its dispersion *in* isopropyl alcohol (IPA) and vacuum filtration. Inset shows the schematic diagram of the sensing-device structure (chemiresistor) realized on paper (substrate) using SWCNT (sensing material) and silver paste (contact). Reprinted with permission from [66]. Copyright 2015: Elsevier. (**b**) Variation in electrical properties of paper with different levels of graphene nanoplatelet (GNP) loading during vacuum filtration. Reprinted with permission from [61]. Copyright 2020: Elsevier.

The analysis of the presented results indicates that these sensors have (i) unique electrical and mechanical properties, (ii) high aspect ratios, and (iii) nanoscale structures due to carbon-based nanomaterials. However, they exhibit sensitivity that is significantly inferior to those of sensors employing paper only. This difference is usually explained by the inherent hydrophobicity of carbon nanomaterials and the very high conductivity of these materials. As shown in Figure 14, the deposition of carbon-based materials, such as CNTs and graphene, on the paper surface results in a sharp drop in the sheet resistance. Another disadvantage of CNTs and graphene is their tendency to agglomerate due to strong van der Waals forces between CNTs or graphene in aqueous solutions [56].

**Figure 15:**
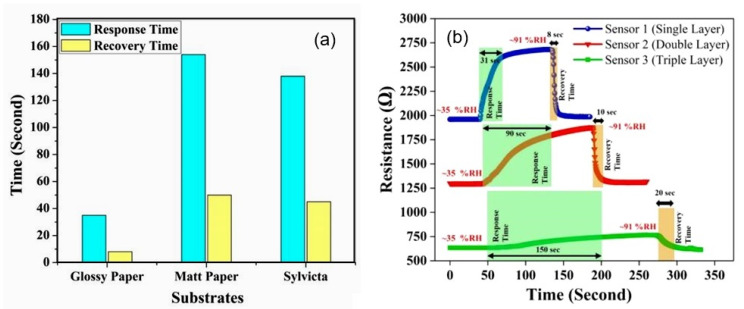
(**a**) Comparative analysis of humidity-sensing characteristics in range of ~35% RH–~90% RH on glossy paper, matt paper, and sylvicta substrates in terms of response/recovery times. (**b**) Influence of the thickness of graphene–carbon layer on the kinetics of sensor response: Sensor 1—thickness 10 µm; Sensor 2—15 µm; Sensor 3—23 µm. Reprinted with permission from [63]. Copyright 2023: Elsevier.

Studies have shown that the characteristics of conductometric humidity sensors, as well as capacitive humidity sensors, depend on the paper used. Beniwal et al. [63] compared the performances of screen-printed graphene–carbon-based humidity sensors fabricated on substrates such as glossy photo paper (150 gsm), matt-sided photopaper (240 gsm), and sylvicta (220 gsm), which is a translucent, functional-barrier paper. They found that all types of paper can be used to develop humidity sensors. However, the sensors printed on matt paper and sylvicta substrates were excessively slow compared to glossy-paper sensors (Figure 15a). Slow response/recovery times maybe suitable for applications such as environmental monitoring or packaging, in which changes in humidity are generally not particularly fast and tend to be gradual [36]. However, they are not desirable for applications such as respiration-rate monitoring, in which short response/recovery times are crucial to easily distinguish between breathing-out/in cycles. This is why, for applications in which fast responses are required, glossy-paper-based sensors are preferable. The functional properties of glossy paper are provided by a special coating, a mixture of materials or polymers, which prevents the penetration of water vapor into the substrate. Furthermore, Beniwal et al. [63] showed that excessive sensing-layer thickness resulted in a marked increase in response and recovery times (Figure 15b).

**Figure 16:**
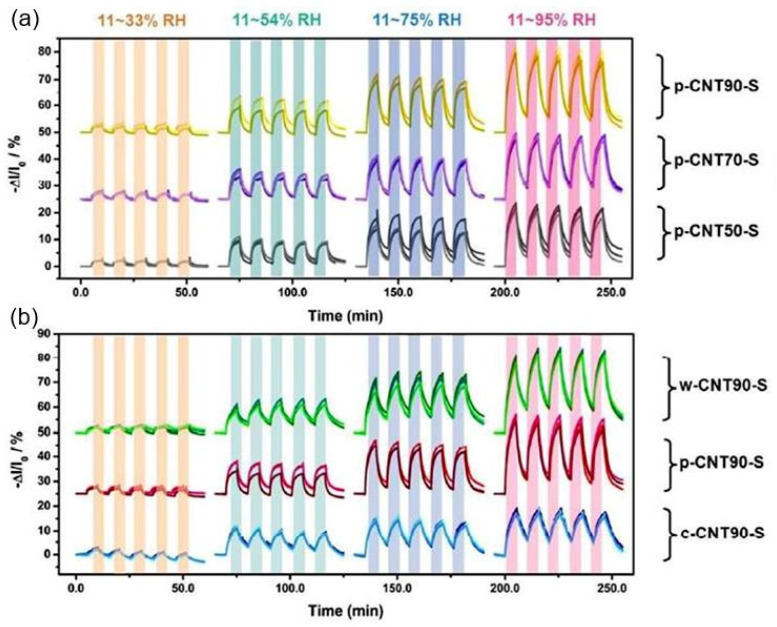
Dynamic-sensing response vs. RH relationship of sensors based on multi-walled carbon nanotubes (o-MWCNTs) with (**a**) different oxidation levels and (**b**) various types of paper substrate. The duration was 300 s for each adsorption or desorption process, and the overlay curves were tested by three sensors fabricated under the same conditions. The p—printing paper, w—weighing paper, and c—cardboard. The 50/70/90 stands for the treatment temperature of MWCNTs during oxidization process. Reprinted with permission from [51]. Copyright 2017: ACS.

Recently, many efforts have been undertaken to improve the sensitivity of carbon-based humidity sensors. In particular, treating CNTs with a mixture of acids, such as nitric and sulfuric acids, oxidization, or plasma processing to increase their hydrophilicity have become universal methods for increasing the sensitivity of sensors based on CNTs to H_2_O vapors. The effects of the paper type and CNT-oxidation mode on sensor performance are presented in Figure 16. Experiments have shown that the characteristics of sensors made of CNTs with higher levels of oxidation yield a higher rate of response and more linear sensor responses as a function of RH changes. However, the observed improvements in sensor performance are very limited [51,60]. In addition, the weak interaction between the CNTs causes poor mechanical stability in the conductive network, which leads to the insufficient durability of paper-based sensors subjected to bending. As a result, a low-cost and easily scalable strategy for fabricating flexible carbon-based humidity sensors with high sensitivity and good durability remains a challenge.

Chu et al. [65] proposed the use of chemical treatment with ethanol to improve the detection of humidity by sensors using graphite as the sensing material. Pencil marks on paper were treated with ethanol to increase the hydrophilicity of the surface. In these studies, it was assumed that ethanol treatment reduces the amount of hydrocarbon on the surfaces of graphite particles. The results obtained by Choo et al. [65] demonstrated that ethanol-treated sensors have better responses to humidity, and that their sensitivity to humidity is directly proportional to their resistance. However, was found that the parameters of these sensors were not stable over time. The ethanol-treated sensor showed a higher level of degradation in its response (~10.5% over 14 days) than the untreated sensor (1.8%).

As a result of the research, it was found that a more effective approach to optimizing the parameters of carbon-based humidity sensors is the application of composite structures. In particular, it was found that the formation of composites based on CNTs and cellulose nanofibers (CNFs) or nanofibrillated cellulose (NFC) can improve the performances of carbon-based sensors [54,56]. For instance, the humidity sensors based on CNTs/CNF composites developed by Zhu et al. [54] had superior sensory responses to those based on CNTs [55]. The excellent affinity of the CNF compared with water vapors made it possible to increase the H_2_O sensitivity of the sensor, albeit to a limited degree.

**Figure 17:**
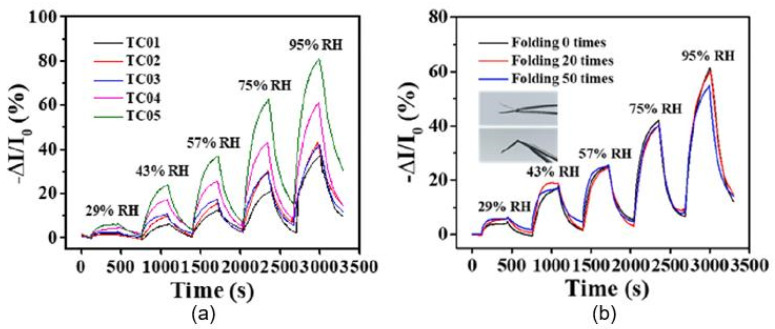
Characterization of humidity-sensitivity performance of the TOCF/CNT-based paper sensors. (**a**) Dynamic-response and -recovery curves of the paper sensors with various TOCF-to-CNT ratios, between 11% and 29, 43, 57, 75, and 95% RH. TC01—TOCF/CNT ratio = 10:1, TC02—15:1, TC03—20:1, TC04—25:1, and TC05—30:1. (**b**) Dynamic-response and -recovery curves of TC04 after folding for different numbers of times. The inset illustrates the folding treatment. Reprinted with permission from [55]. Copyright 2021: Elsevier.

A further study by Zhu et al. [55] showed that the use of conductive 2,2,6,6-tetramethylpiperidine-1-oxyl (TEMPO)-oxidized cellulose fibers (TOCFs) in a CNF/CNT composite greatly improved the sensitivity of humidity sensors. The large number of hydrophilic hydroxyl groups on the surfaces of TOCFs provides more adsorption sites for water molecules and facilitates the transfer of electrons from TEMPO CNTs. The swelling of TOCFs can also increase the sensory response to humidity by destroying the conductive network of CNTs, which determines the resistance of the sensor. The characteristics of these sensors are given in Figure 17a. It can be observed that increasing the TOCF content in the detector material improves the sensory response. However, the authors found that the baseline dynamic-response curves of sensors with high concentrations of TOCFs in the CNF/CNT composite exhibited an obvious drift. The authors believe that the drift in the sensors arose from the fact that they have low recovery rate compared to their response rate. Water molecules built up on the surfaces of materials during the recovery process, resulting in the appearance of unbalanced water-adsorption and-desorption cycles in the form of a gradual increase in the current baseline. With further reductions in the contents of CNTs in the CNF/CNT composite, the conductivity of the composite sharply decreased. As a result, the current signal became excessively small, and the response characteristic of the p-type-semiconductor CNTs disappeared, indicating that the sample was an insulator.

According to Zhu et al. [54,55,56], the main advantages of CNT/CNF- and CNT/TOCF-based humidity sensors are their (i) excellent linearity, (ii) good stability, for more than 15–30 days, (iii) outstanding flexibility, with a maximum curvature of 22.2 cm^−1^, and (iv) folding resistance, up to at least 50 times. As shown in Figure 17b, the response of the sensor shows only a minor change before and after bending/folding, indicating that the sensors are highly resistant to such mechanical stresses. The low sensitivity of the sensors to mechanical impact, their low resistance, and the linear dependence of the sensor signal on the humidity concentration greatly facilitate the measurement process. However, these sensors cannot be considered high-speed sensors, since the estimated response/recovery times are 333/523 s, respectively. These are still relatively slow response and recovery times. When comparing the sensory-response kinetics of the CNF/CNT sensors developed by Zhu et al. [54,55] and the sensors based on CNF/GNP composites developed in [62], the latter sensors, fabricated over a plastic PEN substrate, have significantly faster response and recovery times, although these are not supported by higher sensitivity compared with the CNF/CNT-based sensors fabricated on a paper substrate. The observed response and recovery times were 17 s and 22 s, respectively. This suggests that the main reason for the slow response and recovery of CNF/CNT- and CNT/TOCF-based humidity sensors is the paper substrate, which can control the process of adsorption and desorption of water vapor due to its high resistance, but does not affect the magnitude of the sensory response. Therefore, paper might be considered as a kind of reservoir for H_2_O molecules. The presence of an additional sensitive layer on the surface of the paper substrate slows down this process even further. This conclusion is consistent with the observations above regarding the development of PB sensors with additional humidity-sensitive materials, according to which the simplest way to reduce the response/recovery time is to passivate the paper surface before applying the humidity-sensitive layer. This passivation layer should isolate the paper from contact with the atmosphere. Various methods can be used in this processing to supply the paper with superhydrophobic properties, such as laser ablation [67], the use of plasma-induced polymerization to create hydrophobic polymer chains on the paper surface [68], coating with organic or inorganic nanoparticles [69,70], and others [71,72].

**Figure 18:**
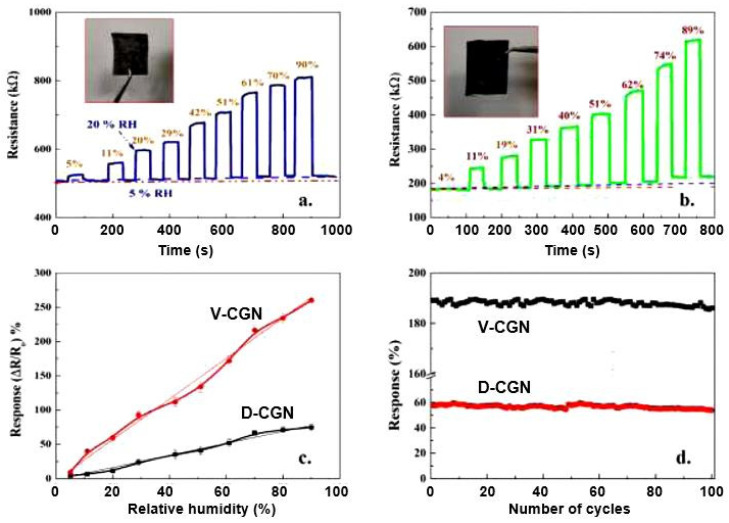
Performance of CGN-based humidity sensors. (**a**) Response and recovery curves of D-CGN-based sensor. (**b**) Response and recovery curves of V-CGN-based sensor. (**c**) Responses of D-CGN- and V-CGN-based sensors. (**d**) Durability and repeatability of the sensor for 100 bending cycles. Reprinted with permission from [61]. Copyright 2020: Elsevier.

It is interesting that humidity sensors based on graphene nanoplatelets (GNPs) have a much higher sensitivity to moisture than CNT-based sensors [61]. The characteristics of these sensors are provided in Figure 18, which demonstrates that GNP-based sensors are sensitive to air humidity in a very wide range, of 5–90% RH, while the sensory response, ΔR/R_0_, is linear, reaching 250% (Figure 18c), and the response and recovery times are only 9–12 s and 15–20 s, respectively. These are the best parameters reported for carbon-based humidity sensors made on paper substrates. In addition, the sensors exhibit excellent repeatability; their response does not change, even after 100 bending cycles (Figure 18d). Furthermore, it is worth noting that a significant increase in the sensory responses of GNP-based humidity sensors compared to CNT-based sensors was obtained using the vacuum-filtration method (V-CGN) in the course of sensor-fabrication process. When using the dip-coating method (D-CGN), this phenomenon was not as noticeable. Apparently, the vacuum filtration, which provides deeper penetration of GNPs into paper and better dispersion, encourages the development of an extended percolation network and the formation of a more optimal interface between GNPs and cellulose fibers. Furthermore, the dip-coating method mainly yields layers of GNPs on the surface of the substrate paper, which prevents the electronic exchange of GNPs with cellulose fibers. Regarding the fast response of humidity sensors based on GNPs using paper substrates, the authors do not explain the reason for the large difference between the kinetics of the sensory responses of humidity sensors based on GNPs and CNTs.

### 3.3. Paper-Based Resistive Humidity Sensors with Solid-State Sensing Materials

The comparative data from the tests of resistive humidity sensors that employ solid materials as the detector elements are brought together in Table 5. It can be seen that only a few studies have been carried out in this direction. However, these studies yielded interesting results.

**Figure 19:**
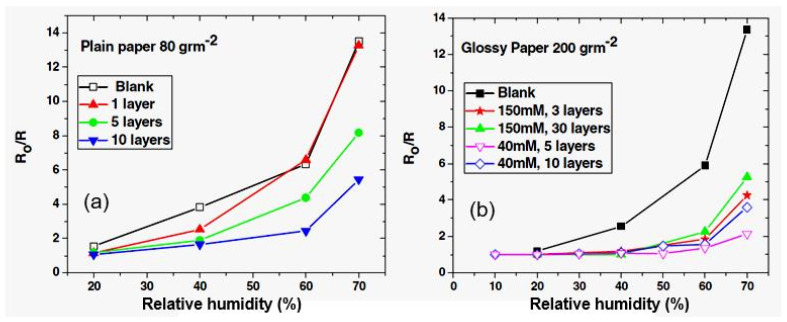
Ratio of electrical resistance to RH levels of 20–70% for various numbers of coatings on the (**a**) 80-grm^−2^ and (**b**) the 200-grm^−2^ paper substrate. Reprinted from [41]. Published 2016 by Elsevier as open access.

It can be seen that the conductometric responses of sensors can vary over a very wide range, depending on the material used and the method of processing. Sensors using CdS [73] and ZnO [41,74] NPs have the lowest sensitivity. Moreover, ZnO NPs on the paper surface have been found to act as passivating coating, reducing the sensitivity of paper-based sensors to air humidity (see Figure 19). Moreover, this effect is most clearly pronounced when applying ZnO NPs to the surface of glossy paper (Figure 19b). The sensors without ZnO coating had a stronger response than the sensors with this layer. The reason for this behavior should be sought in the extremely high resistance of ZnO films. In this case, the resistance of the sensor and, hence, its conductometric response, is controlled not by the ZnO layer, but by the paper substrate. The ZnO layer acts only as a surface-membrane filter, preventing the penetration of the paper by H_2_O molecules. For the conductivity of ZnO to efficiently control the resistance of the sensors, it is necessary to significantly reduce the resistance of ZnO, which can be achieved through doping or by changing the deposition technology.

**Figure 20:**
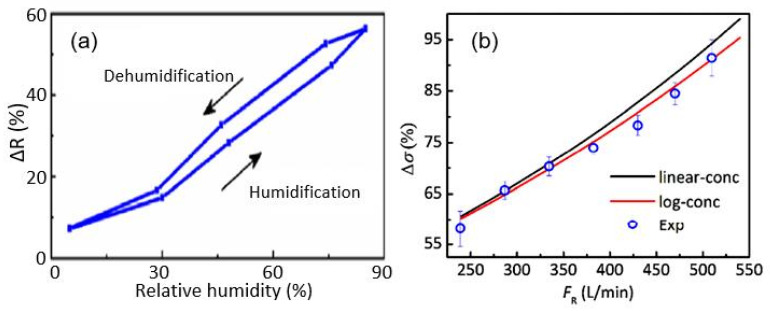
(**a**) Dependence of relative changes in the resistance of the CdS-based sensor fabricated on filter paper with Ag electrodes on the RH. The CdS layer had a thickness ~10–15 µm. (**b**) The variation in the electrical conductivity with the flow rate of humid gas (F_R_). Reprinted with permission from [73]. Copyright 2019: Elsevier.

**Figure 21:**
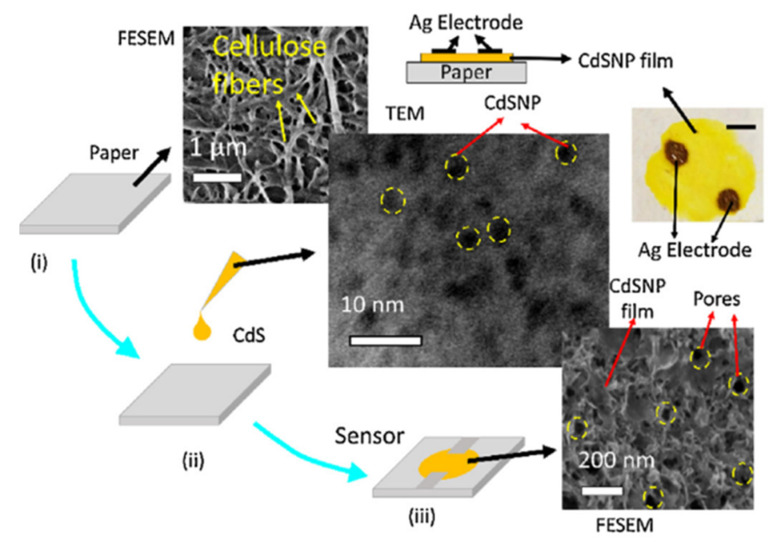
This figure shows fabrication steps (i–iii) of the sensor, along with the FESEM image of the paper surface, TEM image of the CdS NPs, a cross-sectional schematic diagram, along with a photographic image of the sensor, and a FESEM image of the paper embedded with CdS NPs. The scale bar in the sensor image measures 1.5 mm. Reprinted with permission from [73]. Copyright 2019: Elsevier.

Humidity sensors using CdS NPs [73,75] are sensitive to H_2_O in a wider range of relative humidity, albeit with a rather low response (see Figure 20a). It was also found that the sensors are stable up to ca. 2 months, with changes in the sensor response of up to approximately 7%. In addition, the authors established that increasing the air flow in the measuring chamber, which had a volume of 1l, significantly increases the magnitude of the sensory response (Figure 20b). Unfortunately, the authors did not indicate the real resistance of the produced sensors. Therefore, the mechanisms controlling the conductivity of the sensors, as well as the resistance of the paper substrate and that of the CdS layer, are unknown. This prevents the formation of unambiguous conclusions about the processes that manage the sensory response. It can only be assumed that the observed behavior of the sensors was due to the influence of the paper used as the substrate and the design errors in the manufactured sensors. In particular, as shown in Figure 21, due to the special structure of paper, CdS nanoparticles do not form the continuous layer necessary to create a channel for the flow of charge carriers, which is determined only by the properties of the CdS NP layer. This means that the conductivity of the sensor appears to be defined by the processes occurring in the paper. Therefore, the role of CdS NPs in this process can be reduced to their effect on the porosity of the paper itself.

**Figure 22:**
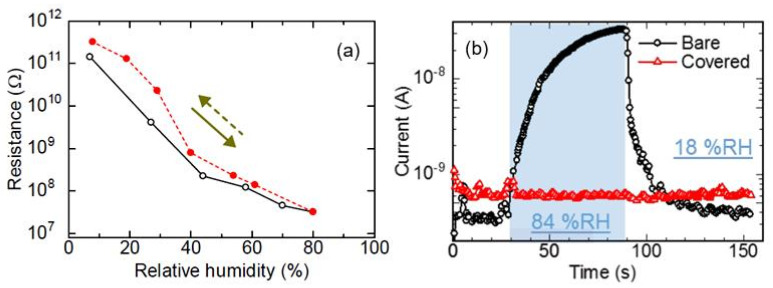
(**a**) Resistance of an all-painted humidity sensor as a function of RH. Open and filled marks represent measurements of increasing and decreasing humidity, respectively. (**b**) Current response to humidity change in the SiO_2_ NP films on cellulose-acetate substrate. Colored region indicates that the films are in 84% RH. 1—uncoated SiO_2_ NP films; 2—SiO_2_ NP films covered by tape. A bias voltage (10 V) was applied to device. Reprinted with permission from [72]. Copyright 2018: ACS.

In principle, SiO_2_ NPs deposited on the surfaces of paper should not control the conductometric sensory response, since SiO_2_ is a good dielectric. However, Kano et al. [72] developed a very highly sensitive humidity sensor based on SiO_2_ NPs with R_0_/R~10^4^ in the range of 10–93% RH (Figure 22a), with an acceptable response rate (Figure 22b). The reaction/recovery times were 31/7 s, respectively. However, it seems that these sensors were not fabricated on cellulose paper, but rather on a single-sided mending tape (3M, Scotch Magic Tape) based on cellulose-acetate foil. Graphite electrodes were formed on the tape using a mechanical pencil with a HB lead and a metal mask. When using photocopy paper as the substrate, the sensor response was much lower and slower; the response/recovery times were 535/87 s. The high sensitivity of the sensors developed by Kano et al. [72] appears to have been due to the influence of the integral effect of the action of water vapor and the interaction between the SiO_2_ NPs and the cellulose-acetate foil. In an acetate film, acetyl (CH_3_CO) groups are attached to long molecular chains of cellulose. Upon exposure to H_2_O, the molecular bonds in these acetyl groups are broken, releasing acetic acid [76]. This seems to be a reason behind the increased responses of sensors based on these substrates. The important role of SiO_2_ in this process is indicated by the fact that the current magnitude observed without the SiO_2_ NPs film is 30 times less than that with the SiO_2_ NPs film, at a relative humidity of 40%. Kano et al. [72] showed that the current-voltage characteristics do not deteriorate after exposure to a relative humidity of 93%. It was also found that the resistance of the sensor increases with higher numbers of bending cycles due to possible damage to the SiO_2_ NP film under bending. However, the sensor remained functional even after a 500-fold bending test at r = 1.3 mm.

### 3.4. Paper-Based Resistive Humidity Sensors with Polymer Sensing Layer

The characteristics of paper-based humidity sensors with polymer detector elements are listed in Table 6.

It can be seen that most of the studies were carried out using polyaniline (PANI) [77,82]. Among various conductive polymers (CPs), polyaniline is widely used due to its stability, ability to control particle size, and ability to form hybrid structures. Compared to CPs, such as polypyrrole, polycarbazoles, polythiophenes, and phthalocyanines, PANI is also of particular interest due to its unique ability to regulate redox conductivity, along with its relatively high achievable electrical conductivity [85,86,87]. The electrical properties of PANI can be modulated by changing its oxidation state and protonating the amino group [88]. When exposed to H_2_O vapors, the effective resistance of PANI films changes due to the appearance of hydrogen bonds between the water molecules and nitrogen present in the amino group [89]. In addition to PANI, PEDOT [83] and polypyrrole [84] were tested to manufacture PB humidity sensors.

**Figure 23:**
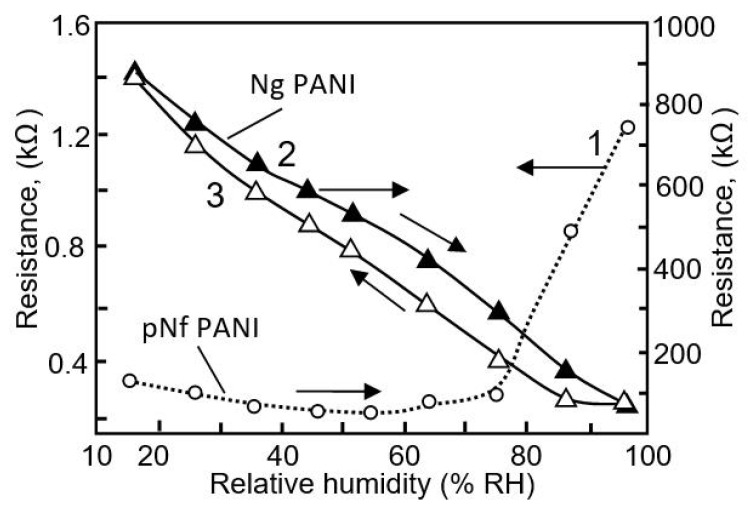
Resistance versus % RH-response characteristics of the (1) pNf PANI- and (2,3) Ng PANI-paper composite-based humidity sensors. Data extracted from [82].

Studies have shown that polymer-based humidity sensors exhibit higher sensitivity than those based on carbon-based materials (see, for comparison, Table 4 and Table 6). However, it was found that the performances of polymer-based sensors depends to a greater extent not on the type of polymer used but on the synthesis technology and morphological features of the applied material. As can be seen from the results shown in Table 6, when using the same polymer, even the parameters of the sensors differ dramatically [86]. The strength of this effect is indicated by the results of the testing of humidity sensors based on nanogranular (Ng) PANI and nanofibrous (Nf) PANI [82]. While the Nf, PANI-based, PB humidity sensors exhibited a low resistance and a bimodal response at higher humidity, of ≥55% RH, the Ng, PANI-based, PB sensors had higher resistance and gave a unimodal linear humidity response over the entire monitored range of humidity, 16–96% RH (Figure 23). This strong difference between the behaviors of the sensors was due to the fact that Nf PANI, in contrast to Ng PANI, exerts a clear polymer-swelling effect. It is known that polymer swelling adversely affects the electrical conductivity of hygroscopic polymer networks limiting the mobility of charge carriers [86].

Regarding the differences between the response and recovery times, which for some PB humidity sensors are in the range of 18–35 s, and are in the range of 1300–2800 s for others (see Table 6), the reasons behind these behaviors in various sensors are not yet known. It is impossible to explain them through the influence of the paper substrate because consistent results were not observed in the relevant studies. It can only be assumed that the slow responses were the result of incorrect measurements due to the employment of overly bulky measurement chambers, or of the influence of the low porosity and high thickness of the polymer coating [90]. For example, Ragazzini et al. [79] used a measuring chamber with a volume of 111,000 cm^3^ to test their sensors. When using such a volume, in which a rapid change in analyte content is difficult, slow sensor response/recovery are quite natural.

**Figure 24:**
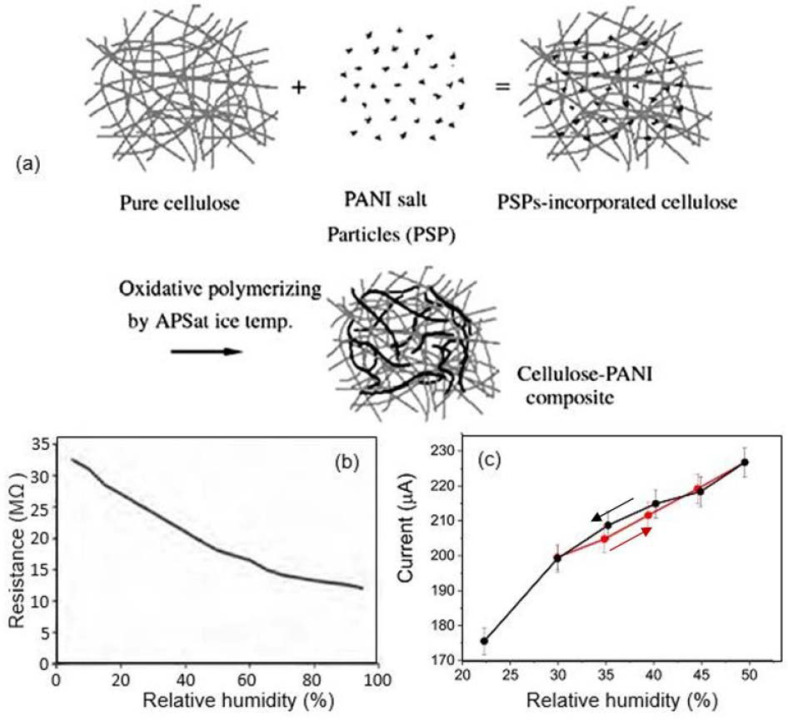
(**a**) Schematic diagram of the process of preparing cellulose–PANI composite. Reprinted with permission from [93]. Copyright 2009: Elsevier. (**b**) Change in resistance of PANI–cellulose with humidity. Reprinted from [78]. Published 2012 by IJEMS as open access. (**c**) Humidity-hysteresis-characteristic curve of cellulose–PANI evaluated in a climate chamber (U = 0.1 V, T = 21 °C). The experiments (*n* = 3) were carried out in a climate chamber with a volume of 111,000 cm^3^ volume, in air, by applying a bias voltage of 0.100 V at stable temperature of 21 °C. Reprinted with permission from [79]. Copyright 2022: RSC.

Many scholars analyzing the results of using PANI in the research on and development of humidity sensors have argued that PANI-based sensors demonstrate poor mechanical strength, reproducibility, and stability under repeated exposure to H_2_O [91,92]. Therefore, attempts have been made to improve the performances of PANI-based humidity sensors by designing nanocomposites such as PANI–CMC [78], PANI–CNF [79], and PANI–SLS [80]. The employment of cellulose, such as CMC and CNF, to create composites is favorable because PANI and cellulose interact strongly due to the presence of hydrogen bonds between the hydroxyl groups in cellulose and the repetitive N-functionality in polyanilines, which contributes to the modification of paper and the production of composite materials with improved properties [87]. The preparation of cellulose–PANI composites is more comprehensively described by the schematic representation in Figure 24a. Mo et al. [93] found that acids are capable of successfully activating cellulose during polymerization and improving the availability and reactivity of O–H groups. Their study showed that the mechanical properties of sensors based on PANI composites are indeed improved. According to Mo et al. [93], the composites were more stable than the pure cellulose due to the protection offered by the PANI. However, the use of PANI–CMC and PANI–CNF nanocomposites did not produce any real improvements in the functional parameters of the humidity sensors. For instance, the effect of exposure to air on the resistance of the sensors based on PANI–CMC composites is displayed in Figure 24b. The resistance of the sensors continuously decreased from ca. 32 MΩ to ca. 10 MΩ as the relative humidity increased in the range of 5–90% [78]. These sensors were stable for over 60 days.

**Figure 25:**
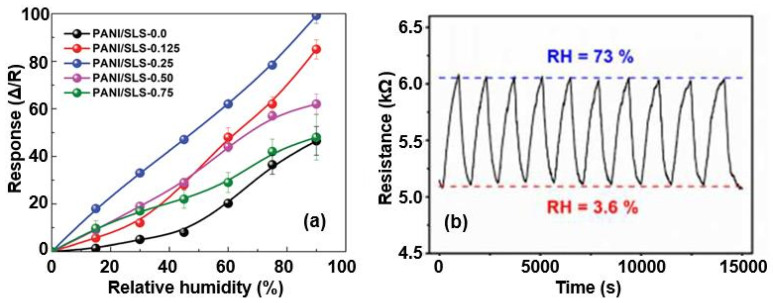
(**a**) Response of the PANI–SLS-X-coated paper sensor as a function of relative humidity. Concentration of SLS changed from 0.0 to 0.75 g. Reprinted with permission from [80]. Copyright 2022: RSC. (**b**) The reversible response of the PPy-based device obtained by alternating RH between two levels (3.6 and 73%). Reprinted with permission from [84]. Copyright 2018: ACS.

Furthermore, PB humidity sensors using a PANI–SLS composite demonstrated improved sensor performance (Figure 25a): the PANI/SLS-based sensors were more sensitive and faster. For comparison, in the case of the pristine PANI, the sensor response, ΔR/R, was 70%, while it increased up to 459% and 588%, with the sensors based on the PANI–SLS (0.125) and the PANI–SLS (0.25), respectively. Mahlknecht et al. [80] showed that an anionic surfactant, such as SLS, is able to control the morphology, electrical properties, and crystallinity of PANI, which can be useful for the operation of humidity sensors. In particular, it was found that PANI treated with SLS surfactants encourages the formation of PANI nanorods, whose diameters are smaller than those of pure PANI nanorods. In addition, the electrical conductivity of PANI–SLS nanorods increased by a factor of three with the addition of SLS. The presence of SLS also prevents the thermal degradation of PANI nanorods. The PANI–SLS-coated paper-based humidity sensor also exhibited excellent repeatability and resistance to multiple bending. These are important results for flexible sensors.

Given the excellent mechanical stability of the PPy structure, attempts have been undertaken to use PPy in flexible humidity sensors. Santiago et al. [84] showed that the use of PPy, subject to a special chemical modification to remove excess chloride ions upon a controlled washing step in water, can significantly improve sensory responses to changes in air humidity. A typical transient resistance of a PPy-based sensor under varying levels of humidity is shown in Figure 25b. The PPy was in situ polymerized on the surface of porous filter paper. The performance of the polypropylene-based humidity sensors was very stable under mechanical-stress testing, withstanding over 600 bending cycles with a loss of sensor response of about 5%. According to the authors, the sensor responses of PPy-based devices are controlled by the swelling effect.

**Figure 26:**
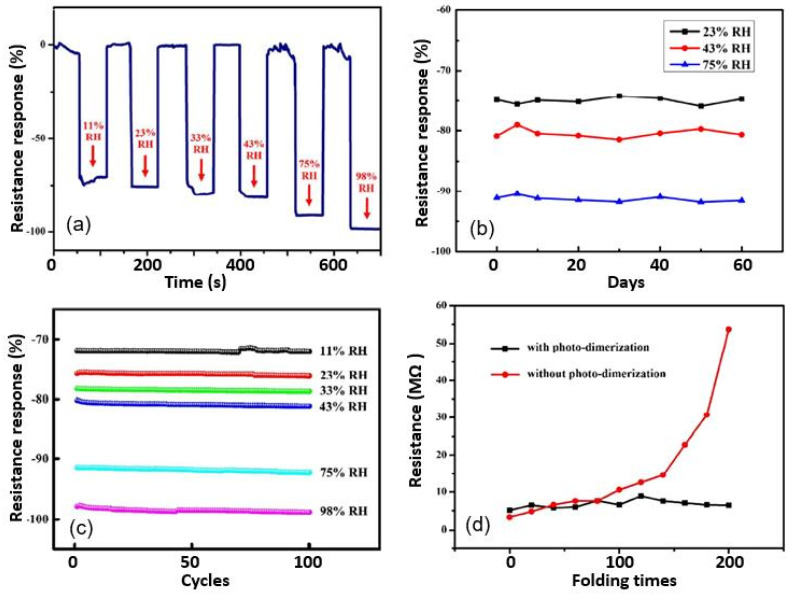
(**a**) Resistance-response measurement of the PEDOT:PVMA sensor under switching RH. (**b**) Long-term stability of PEDOT:PVMA sensor exposed to 23%, 43%, and 75% RH. (**c**) The resistance of sensors with different folding times. (**d**) Influence of photo-dimerization on the stability of PEDOT:PVMA-film resistance during multiple folding. For these measurements, 50-layer PEDOT:PVMA sensors that were photo-dimerized for 6 h were prepared. Reprinted with permission from [83]. Copyright 2016: RSC.

Yuan et al. [83] found that PEDOT:PVMA-based humidity sensors are also promising candidates for use in various applications. The PVMA was used as a soft template to achieve the oxidative polymerization of the PEDOT. Simultaneously, the PVMA acted as a PEDOT dopant, encouraging the aqueous dispersion of PEDOT:PVMA. The cross-linked PEDOT:PVMA films were transferred onto a paper substrate by using inkjet printing. The developed sensors were fast and sensitive to changes in humidity over a wide range (Figure 26a). In addition, the parameters of the sensors had acceptable stability (Figure 26b). The sensory response did not change dramatically under normal relative-humidity conditions for at least 60 days. Their performance was also not strongly affected by substrate bending. The functional characteristics were retained even after 100–200 bending cycles (Figure 26c). All of these results were achieved by employing PVMA photo-dimerism. The photo-dimerization of PVMA via the creation of dimers through a photochemical reaction made it possible to transform the cross-linked film into a stable and highly flexible layer. As shown in Figure 26d, the resistance of the photo-dimerized sensors was stable in the range of 3–8 MΩ under all the bending angles applied, while the resistance of the sensors without photo-dimerization sharply increased to 54 MΩ, under the same conditions. The authors suggested that the photo-dimerization of PVMA upon irradiation with UV light at a wavelength of 365 nm transformed the cross-linked structure, making the conductive films significantly denser.

## 4. Impedance Paper-Based Humidity Sensors

Despite the relatively complex process through which impedance-based sensors are measured, they still detect humidity efficiently. As indicated above, impedance is conventionally measured in the 100-Hz–1-MHz range using an impedance analyzer. There are currently several reports on the development of such impedance-based PB humidity sensors. Their major functional parameters are given in Table 7.

When developing impedance-based humidity sensors, conductive polymers, such as PEDOT:PSS and PANI [89], as well as poly(ionic liquids) (PIL) [96], were used as a humidity-sensitive materials. Poly(ionic liquids) are polyelectrolytes that include cations or anions in each repeating monomer unit of the polymer backbone. Polymerized PILs inherit high ionic conductivity from ionic liquids and good mechanical stability from polymers. Polymers are rather easily applied to paper surfaces by inkjet printing. To prepare the ink, the polymer in ethylene glycol and ultrapure water are employed as a solution. The PIL layer is formed on the paper substrate by drop casting, followed by UV irradiation, with further washing in methanol to remove unreacted monomer residuals.

From the data presented in Table 7, it can be seen that the PEDOT:PSS film printed on paper showed the smallest variation in impedance, with a change in relative humidity from 16% to 90%, less than one order of magnitude. Additionally, PANI-based humidity sensors have the highest sensitivity [89]. Studies show that the presence of water stimulates the protonation of nitrogen atoms in PANI. This effect leads to the appearance of stabilized polycations between the π-delocalization along the polymer chain and increases in the electrical conductivity in the polymer [102]. However, the maximum sensitivity of the sensors is observed at a relative humidity 16–45% and, with further increases in air humidity, the sensitivity drops significantly. Gomez et al. [77] also developed an impedance sensor using PANI. The PANI was applied by inkjet printing on bond and photographic paper. A typical view of the Cole–Cole plots for these sensors is presented in Figure 23a. The Cole–Cole diagrams obtained for the doped PANI on the bond paper showed a characteristic shape with a stretched semi-circle (at high frequencies) followed by a linear region at low frequencies, attributable to the Warburg impedance and other intrinsic mechanisms of conductivity in the PANI, which was used as a porous medium. The experiment showed that bond paper absorbs PANI better photographic paper and provides a more uniform distribution of the PANI over its surface. Unlike the sensors developed by Morais et al. [89], the sensors developed by Gomes et al. [77] exhibited the same sensitivity to H_2_O vapors in the entire range of relative humidity from 10% to 90%. However, their sensitivity was low. The reason for this strong difference between the parameters of the sensors based on PANI was not clarified.

**Figure 27:**
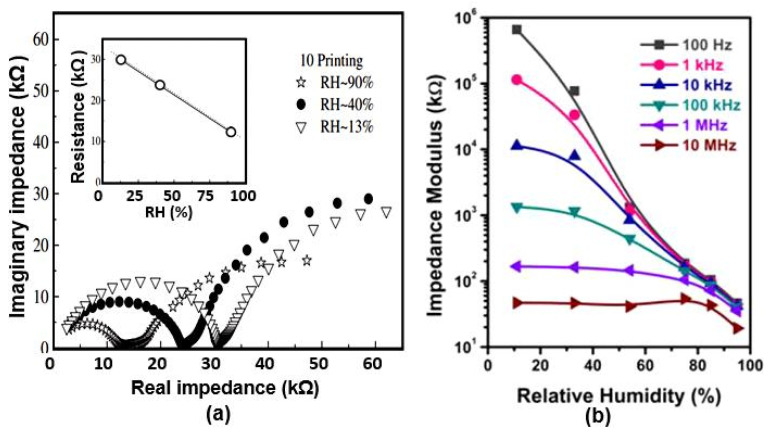
(**a**) Cole–Cole plots for doped PANI printed on bond paper with ten printings for three different values of RH%. Reprinted with permission from [77]. Copyright 2012: Elsevier. (**b**) The impedance modulus vs. RH curves of PILs@Paper5 sensors with pencil-trace electrodes measured under different frequencies. Reprinted with permission from [98]. Copyright 2021: Elsevier.

In this regard, PIL-based sensors behave favorably compared with the sensors considered above because they have a high sensitivity over the entire range of relative humidity, 10–95%. For these PB humidity sensors, the impedance vs. frequency curves are shown in Figure 27b, while Table 8 summarizes the effect of the amount of PIL applied to the paper surface on the sensors’ functional performance. It can be observed that the introduction of fPIL causes the sensors to become more sensitive. In addition, the response rate increases with the higher PIL content. The hysteresis is also reduced with a moderate introduction of PIL. However, increases in hydrophilicity always lead to complications in the desorption process. As a result, the recovery time increases after PIL loading, and the hysteresis becomes larger when the PIL content exceeds 24 wt.%. Taking these regularities into account, Zhao et al. [96] decided that humidity sensors have optimal functional parameters when the PIL content corresponds to 15–24 wt.%. These sensors exhibit the best linearity in terms of the RH–impedance modulus, minimum hysteresis, and acceptable responses to variations in humidity.

As a sensitive material [97,98], or as a component of a composite [95,100], paper can be also employed in impedance humidity sensors. However, it was found that almost all paper-only sensors become sensitive to H_2_O when the relative humidity is higher than 50–60%. Sensors based on ZnO/CNF composites had approximately the same characteristics [100]. Zinc-oxide nanocrystals were grown on cellulose fibers with a simple one-pot aqueous chemical-bath deposition technique using hexamethylenetetramine (HMT) as the surfactant. Despite the high stability of the parameters, the maximum sensitivity was observed only at RH > 80%. In addition, the sensors were very highly resistive, with R_o_ > 10^8^ Ω. In this regard, PANI–CMC composites seem to be more attractive for the development of humidity sensors. According to Kotresh et al. [95], sensors based on PANI–CMC composites were sensitive in the relative humidity range of 25–75%. In addition, these sensors were quite fast. However, these sensors were fabricated on a glass substrate.

**Figure 28:**
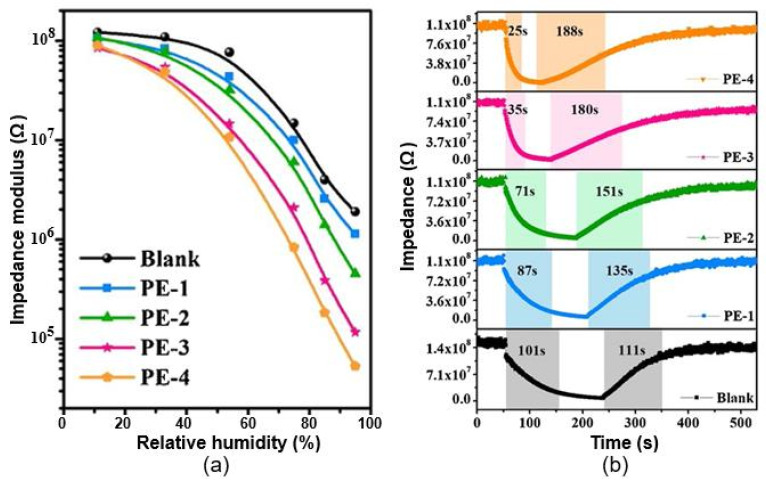
(**a**) Impedance modulus–RH curves of the blank paper and PE-1–PE-4 sensors. (**b**) Response and recovery curves of the blank-paper and PE-1–PE-4 sensors between 11% and 95% RH. Sensors were prepared by changing the reaction amount of the EPTAC. The reaction ratios of EPTAC (NaOH solution with paper: EPTAC) were 0, 1:0.1, 1:0.2, 1:0.5, and 1:2. Sensors with different concentrations of EPTAC are designated as blank, PE-1, PE-2, PE-3, and PE-4. The sensors had Ag interdigitated electrodes. Reprinted with permission from [98]. Copyright 2021: Elsevier.

Some research studies found that it is necessary to functionalize paper to expand a range of relative humidity levels and improve the sensitivity of humidity sensors. In particular, to improve paper’s conductivity, the treatment of paper with a solution of NaCl [97] or NaOH [98] was found to be highly powerful. According to Farrell et al. [103], when alkali and cellulose are mixed in solution to form reactive EPTAC (epoxy propyl trimethylammonium chloride), trimethyl amine is emitted. Subsequently, EPTAC reacts with hydroxyl groups on the cotton fiber under alkaline conditions to form cation-contained cotton. In the presence of alkali, cellulose converts to EPTAC, together with the partial dissociation of the cellulose hydroxyl group [104]. For these purposes, spray coating, dip coating [97], and immersion in solution [98] can be applied. The influence of the reaction ratio between the NaOH solution and the paper on the impedance characteristics of the sensors upon exposure to varying levels of humidity is shown in Figure 28. It can be seen that treatment with the EPTAC solution is similar to the effect of PIL. On one hand, there is an increase in sensory response and a decrease in the response time and, on the other hand, an increase in the recovery time is observable. Due to the characteristics of EPTAC, the free movement of Cl^−^ ions can encourage the electrical conductivity of materials under wet conditions. It was also found that the developed paper-based humidity sensors have good flexibility. The sensor output remained stable under bending conditions, with a slight decrease in sensitivity. As the degree of bend increased, indicated by the reduction in the radius of the curvature, the sensor impedance tended to grow, but this variation was rather minor. Rivadeneyra et al. [99] found that the TEMPO oxidation of cellulose nanofibers (CNF) also contributed to the expansion of the sensitivity range of humidity sensors. They reported that the sensors they developed had high sensitivity over the entire range of relative humidity, from 20 to 85%. According to Rivadeneyra et al. [99], the sensors were also fast (see Table 7).

## 5. Mass-Sensitive, Microwave and Fiber-Optic Paper-Based Humidity Sensors

In addition to the humidity sensors discussed above, other types of sensor have been developed based on paper, such as mass-sensitive, microwave, and fiber-optic sensors [105]. The principles of their operation are described in detail in [9,10]. It should be noted that little research has been carried out on the development of the PB humidity sensors listed above.

### 5.1. Mass-Sensitive PB Humidity Sensors

**Figure 29:**
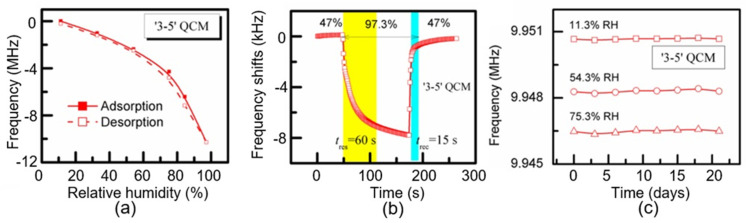
(**a**) Humidity hysteresis of CNC-based QCM humidity sensors. (**b**) Dynamic responses of CNC-based QCM humidity sensors. (**c**) Long-term stability of ‘3-5′ QCM humidity sensors. Reprinted with permission from [106]. Copyright 2020: Elsevier.

Mass-sensitive sensors include quartz-crystal microbalance (QCM) sensors, surface acoustic wave (SAW) sensors, capacitive micromachined ultrasonic transducers (CMUTs), and microcantilevers. Mass-sensitive moisture detection is mainly based on the piezoelectric effect, which converts the change in mass of a humidity-sensitive material after the absorption of water molecules into a change in frequency. The sensitivity of these sensors largely depends on the properties of the paper used as a humidity sensitive material, namely the density of the paper and its adsorption capacity. Most studies consider cellulose nanocrystals (CNC) as humidity-sensitive materials in QCM devices [106,107,108,109]. In particular, the sensors fabricated by Yao et al. [106] also fabricated a quartz-microbalance humidity sensor using a polydopamine (PDA)/CNC/graphene-oxide nanocomposite. Some characteristics of these sensors are shown in Figure 29. The results of the testing of these sensors showed that adding PDA@CNC to a graphene-oxide film can significantly improve the sensitivity of the sensor while maintaining high stability over the entire humidity range. The analysis of the functional characteristics observed in this CNC-based QCM sensor shows that its sensitivity to water vapors can be effectively improved by employing the asymmetric-electrode structure. In addition, the sensor with asymmetric electrodes displayed reduced hysteresis, faster response and recovery, and excellent long-term stability. Wang et al. [110] developed a SAW humidity sensor with bacterial cellulose (BC) as the sensing material. Bacterial cellulose is an environmentally friendly material that is moisture-sensitive, inexpensive and, in particular, commercially available. In addition, the bacterial cellulose film is a highly porous network of ultra-thin interlaced fibers, and its surface contains a large amount of hydroxyl groups, which greatly improves the absorption capacity of the humidity-sensitive layer. Bacterial cellulose was applied to the surface of SAW using the spin-coating method. Wang et al. [110] reported that SAW PB sensors had high sensitivity and fast responses and recovery. Zheng et al. [111] used a nitriding-oxidation wafer-bonding process to fabricate a capacitive CMUT humidity sensor. In this work, a CNC layer was spin-coated onto the top layer of a vibrator as a humidity-sensitive layer. The experimental results showed that the CMUT humidity sensor had high sensitivity, fast recovery, low hysteresis, good reproducibility, and long-term stability. Zheng et al. [111] believe that the CMUT can be a high-performance miniature sensor platform for humidity monitoring. The main parameters of mass-sensitive PB humidity sensors are shown in Table 9.

### 5.2. Fiber-Optic PB Humidity Sensors

**Figure 30:**
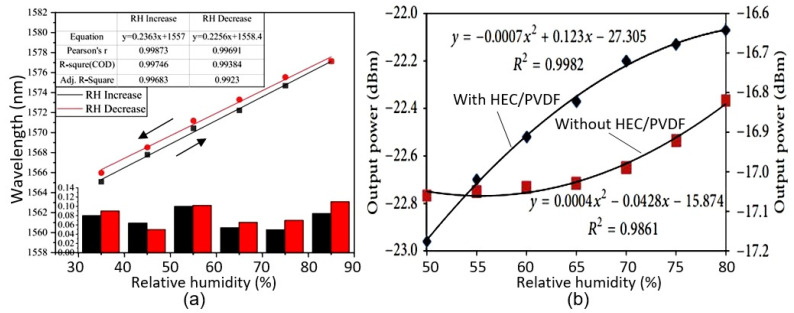
(**a**) The wavelength-resonance changes with RH of CMC/CNT composite film. Reprinted with permission from [118]. Copyright 2020: Elsevier. (**b**) The transmitted light from the silica tapered fiber versus the relative humidity. It can be seen that the sensitivity of the sensor significantly increases when the tapered fiber is coated by the HEC/PVDF composite. Reprinted from [115]. Published 2013 by Hindawi as open access.

In principle, fiber-optic humidity sensors should be of increased interest because fiber sensors are cheaper than conventional electrical sensors and better suited to use in hazardous or explosive environments, or when immunity to electromagnetic interference is required [113]. They also offer the ability to multiplex a large variety of sensors (temperature, displacement, pressure, pH, humidity, etc.) onto the same optical fiber, reducing the need for multiple cables/interfaces, as in traditional electronic and electrical-monitoring systems. The sensitive mechanism of fiber-optic sensors is mainly based on changing the parameters of the light beam. In particular, the optical properties, intensity, wavelength, frequency, etc., of the light of fiber-optic sensors are modified when H_2_O vapors interact with a detector material deposited on the surface of the optical fiber [113]. Fiber-optic PB humidity sensors are usually fabricated by coating the surface of an optical fiber with humidity-sensitive materials, such as hydroxyethyl cellulose (HEC) [114,115], carboxymethyl cellulose (CMC) [116], liquid crystalline cellulose (LC) [117], or cellulose-based composites [118] (see Table 10). The detection mechanism is based on expanding the volume of cellulose coated on the optical-fiber surface following water absorption, which leads, in turn, to a shift in the reflection spectrum (Figure 30a) or to a change in the transmitted-light intensity (see Figure 30b). The device’s performance largely depends on the thickness of the paper-based film, the swelling effect, and the water-vapor-diffusion velocity in the film. The content of the composites also has a large impact on the sensory response. For example, according to Li et al. [118], the humidity response was significantly improved after increasing the rational amount of CNTs in a CMC/CNT composite. The response increased by approximately 35%. Due to the low thickness of the detector layer, the sensors were very fast. They were also characterized by stable parameters. The performances of PB fiber-optic-humidity sensors are listed in Table 10.

### 5.3. Microwave PB Humidity Sensors

**Figure 31:**
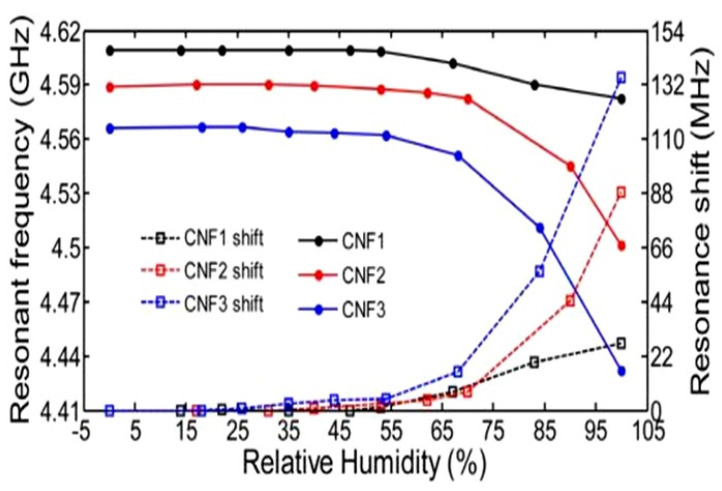
Resonant frequency of CNF films as a function of RH. The CNF 1, CNF 2, and CNF 3 correspond to samples of different sizes, increasing with sample number. Reprinted with permission from [121]. Copyright 2017: Elsevier.

Microwave humidity sensing is based on the detection of dielectric changes with fluctuations in humidity caused by water absorption and increases in the volume of humidity-sensitive materials [121]. Microwave sensors are virtually unaffected by changes in conductivity and can detect internal moisture without damaging the outer packaging. Moisture detection is made possible by shifting the resonant frequency of the device (see Figure 31). This means that achieving a maximum sensory response requires the use of paper with maximum thickness, capable of adsorbing sufficient moisture to significantly change the dielectric constant. For example, Eyebe et al. [121,122] fabricated a series of microwave humidity sensors using CNFs (see Table 11). Tanguy et al. [123] developed a microwave resonator (OMR) humidity sensor using a conductive PEDOT:PSS polymer deposited on a flexible film of cellulose nanofibrils (CNFs). The CNF substrate served as a functional sensor component to give the microwave resonator superior humidity-sensing performance. The sensor operated on the basis of the simultaneous adsorption of moisture by the CNF film and the interaction of water molecules with the PEDOT:PSS. 

## 6. RFID (Radio-Frequency Identification) Humidity Sensor

Radio-frequency identification (RFID) technologies have been applied in many industries to automatically identify and track objects such as packaged goods during storage, handling, and transport in the supply chain. Radio-frequency identification with an additional sensory function (called RFID sensor tags) allow the improved monitoring of goods with more diverse and newer technologies. Therefore, they have received significant attention in both industry and academia [124,125]. One example of the application of a RFID sensor tag is smart packaging [126]. The RFID sensor tag equips traditional packaging with smart features to inform customers as to products’ status, or the environmental conditions under which products are stored [127,128]. These technologies require a variety of cheap sensors, including humidity sensors. Therefore, it is justifiable to aim developments at the fabrication of cheap paper-based RFID humidity sensors [129,130]. In these devices, the humidity sensor can be built into the antenna array, and its detuning can be matched to the appearance of H_2_O vapors by a powerless RFID method [131,132].

**Figure 32:**
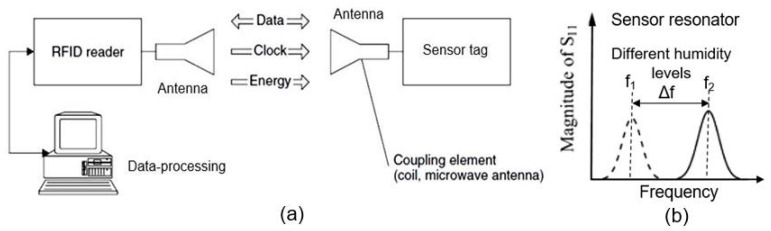
(**a**) Typical RFID system consisting of reader, sensor tag, and data-processing server. (**b**) Operation principle of the chipless RFID humidity sensor based on frequency-spectrum signature.

The structures and principles of operation of RFID sensor tags, as well as the method used to perform humidity measurements, are discussed in detail elsewhere [132]. The most popular units are passive RFID sensor tags, primarily because they do not need a power supply. This means that sensor tags do not need maintenance procedures and can be permanently embedded in walls, ceilings, or floors for a long-term monitoring even for several years. At the beginning of communication, the reader sends a continuous unmodulated carrier wave, which is received by the tag’s antenna (see Figure 32a). The incoming signal is routed to the IC chip, which uses its internal rectifier to convert the weak AC to DC. The internal voltage doubles are then used to charge the capacitor to act as energy storage, allowing short periods of operation without the presence of a carrier wave. Once the capacitor is charged to a sufficiently high DC voltage, the sensor tag is activated and becomes ready to receive commands from the reader. The reader receives information about the state of the object and the ambient air humidity through a change in the frequency of the LC resonator, which includes a humidity sensor (see Figure 32b).

**Figure 33:**
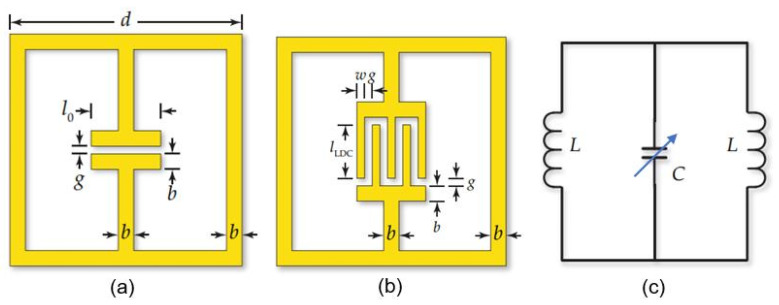
Schematic of the layout of a planar electric LC resonator. (**a**,**b**) A typical ELC resonator is composed of a central capacitive gap connected to two inductive loops. (**c**) An equivalent circuit of the resonator constitutes an LC resonator (the resistance is neglected here). Reprinted from [134]. Published 2020 by OSA as open access.

As sensitive elements in RFID sensor tags, electric LC-resonators are ordinarily employed; a typical structure of these resonators is shown in Figure 33. There are several examples of research on and development of RFID sensor tags with planar LC resonators for humidity detection. In particular, Feng et al. [133] applied inkjet-printing technology to fabricate a resonator on a paper substrate, using Ag NP-based ink. The authors compared three types of paper substrate: inorganic coated inkjet paper (PEL Nano P60, Printed Electronics Ltd., Cambridge, UK), commercial inkjet photo paper (Ultra premium, Kodak), and commercial UV-coated packaging paper (Korsnäs AB). The tests showed that the paper-based LC resonators had excellent sensitivity, of ca. 400 kHz/% RH, to variation in air humidity, with an acceptable response time, τ, in the range of 10–15 min. In this case, the sample examined on the packaging paper exhibited the highest sensitivity of the three sensor types at a humidity, below 70% RH. At a relative humidity greater than 70%, the sensory response was significantly lower. Feng et al. [133] believe that using a thinner paper substrate or a higher operating temperature can facilitate the desorption of H_2_O molecules more efficiently if a faster response is required.

**Figure 34:**
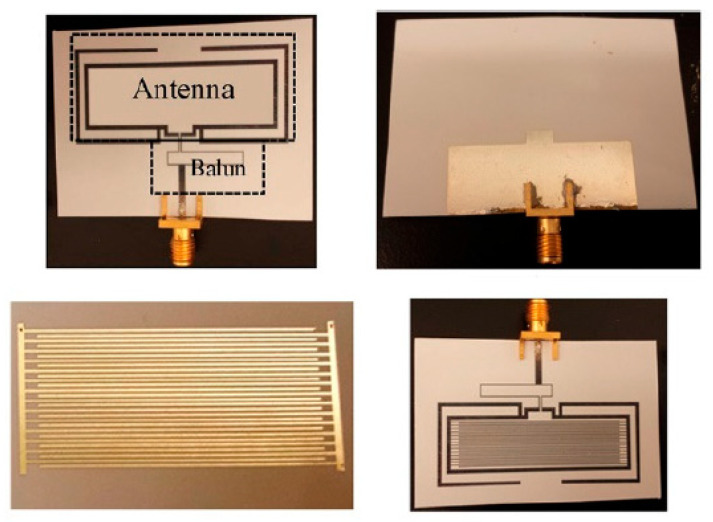
Paper-based resistive humidity sensors: exploitation of substrate-resistivity variations via antenna detuning for a passive RFID sensor node. Reprinted from [135]. Published 2016 by MDPI as open access.

Another variation of the paper-based resistive humidity sensors developed by Quddious et al. [135] for use in in RFID sensor tags presented in Figure 34. The printing of ID electrodes on a paper substrate provided baseline conductivity, subject to humidity changes. Due to the porous nature of the substrate, the variation in the relative humidity from 18% to 88% reduced the resistance of the PB sensor from a few MOhms to kOhms. The response time was about 3 min. Passive wireless sensing was carried out using an antenna, and the internal circuit responded to changes in sensor conductivity in response to the H_2_O input, while the external dipole arm was applied for chipless identification.

The parameters of other RFID humidity-sensor tags are listed in Table 12. It can be seen that the sensors developed by Xie et al. [136] have lower sensitivity but a higher quality factor. To fabricate their RFID humidity sensor, Xie et al. [136] used A4 printing paper (70 g/cm^2^, 88 μm thick) and Ag-based ID electrodes. The area of the designed sensor was 2 × 2 cm^2^. The width and the spacing of the ID electrodes were both 300 μm. A copper coil, 2 cm in diameter, was used as a readout antenna.

## 7. PB Humidity Sensors Based on Shape Deformation and Luminescence

In the literature, there are further examples of the design of humidity sensors beyond those reviewed above. Here, we survey some of the more remarkable cases, which are worthy of discussion.

### 7.1. Shape-Deformation-Based PB Humidity Sensors

One of the non-traditional approaches to the development of PB humidity sensors is the use of shape deformation under the influence of moisture [44,138]. The sensing elements of these sensors normally consist of a thin moisture-sensitive film. The key feature is that the two sides of the humidity-sensitive film have different coefficients of expansion when in contact with moisture or water-vapor absorption, which leads to the bending of the film as a result of its asymmetric expansion under varying levels of humidity [139]. Indeed, typical films with symmetrical structures cannot bend due to the symmetrical changes in their volume under the influence of humidity. Therefore, the humidity sensors with the highest shape deformation have a sensing element consisting of two or more layers of materials with different water-absorption characteristics [140]. However, bending is still possible, even for paper that has the same properties on both sides, if only one side is exposed to the flow of humid air. This option was considered by Wang et al. [141]. They observed that reversible, moisture-controlled bending was possible in an 8-µm-thick nano-fibrillated cellulose (NFS) film; the degree of bending depended on the moisture difference on both sides of the film. Stationary bending was achieved in fractions of a minute. After removing the source of moisture, the film returned to its original position. Wang et al. [141] found that bending is very sensitive to moisture, as shown by the observation that the film bent even when a human hand approached the film at a distance of a few millimeters. The curvature of the bend decreased with the increase in the film thickness. This explains the fact that the effect was not observed in significantly thicker conventional paper sheets.

**Figure 35:**
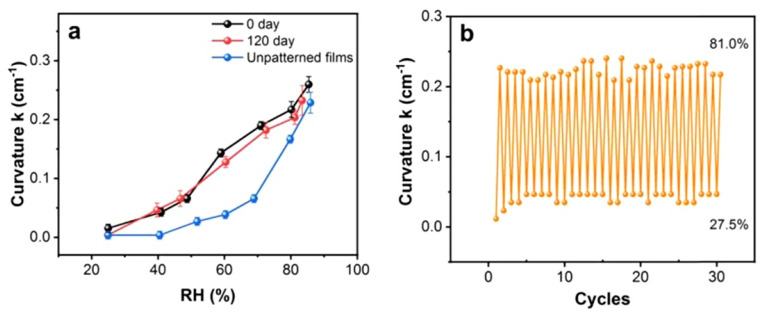
(**a**) Dependence of the curvature of patterned CNF/GO films (freshly prepared and stored for 120 days), as well as unpatterned ones, on humidity. (**b**) Cyclic-response performances of CNF/GO films during RH switching between 27.5 and 81.0%. Reprinted with permission from [146]. Copyright 2020: ACS.

Research has shown that this simple shape-deformation-based humidity-detection method can be implemented using inexpensive paper materials. Various approaches can be used for this purpose. For example, one side of a paper-based cantilever may be coated with a moisture-swellable polymer [138]. The mechanical deflection of the cantilever, caused by the swelling of the polymer, creates a tilt angle characteristic of a certain level of air humidity. One side of the paper can also be treated with cellulose stearoyl ether to make it hydrophobic [142,143]. This prevents the adsorption of water vapor on that side of the paper and causes the cantilever to bend in proportion to the level of humidity. Water adsorption on the hydrophilic side expands the cellulose, while the hydrophobic side changes little, which leads to directional deformation, causing the cantilever to bend. A GO/ethyl cellulose (EC) actuator also responded to changes in air humidity by bending and, therefore, can also be used as an actuator to fabricate shape-deformation humidity sensors [144]. In addition, it was reported that an actuator for a shape-deformation humidity sensor can be fabricated by applying CNC and polyethylene glycol diacrylate (PEGDA) to both sides of a polyamide (PI) film [145]. Cantilever bending in a humid atmosphere can be associated with varying degrees of moisture absorption and expansion of CNC and PEGDA on both sides of a PI film. Li et al. [146] developed a shape-deformation humidity sensor with an ultra-fast response using a cellulose nanofiber (CNF)/graphene oxide (GO) composite film with an asymmetric pattern. Composite CNF/GO films were produced by vacuum filtration followed by surface imprinting. The results showed that the composite films had excellent linear humidity responses and cyclic stability over a range of 25 to 85% RH (see Figure 35). The curvature of the film changed from 0.012 cm^−1^ to 0.260 cm^−1^ when the relative humidity changed from 25% to 85%, and the response time was only 3–5 s. The excellent humidity response was due to the addition of GO, which actively interacted with the water, increasing the flexibility and sensitivity of the composite films to moisture.

Zhou et al. [147] developed moisture-controlled actuators based on carbon nanotube (CNT)-coated paper and a biaxially oriented polypropylene (BOPP) composite. The CNT-paper composite/BOPP actuator exhibited a strong change in curvature with changes in humidity (the average curvature was 1.2 cm^−1^). The thickness of the CNT-paper/BOPP composite film was 76 μm. The CNT-paper/BOPP actuator was manufactured at 60% RH and, therefore, the actuator was not bent at this humidity. When the relative humidity decreased to 14%, the actuator bent towards the CNT-paper. The curvature rapidly enlarged in the first 60 s. When the relative humidity increased to 60%, the CNT-paper/BOPP drive returned to its initial flat state. In addition, the relative humidity continued to enhance rapidly in the first 60 s. Cao et al. [148] showed that a hierarchical gradient structure based on natural bamboo, MXene (Ti_3_C_2_T_x_) and polydopamine (PDA) (G-MXCP) is also sensitive to moisture. To form this structure, a layer-by-layer assembly method was employed, which was based on a vacuum-filtration process. Cao et al. [148] found that the ultra-high tensile strength, the modulus of elasticity, and the superior impact toughness of the G-MXCP nanocomposite, resulting from the mesoscale gradient structure and strong hydrogen-bond interaction, imparted to the G-MXCP actuator high levels of the desired mechanical reliability compared to most adhesive bilayer structural materials. Tests have shown that the manufactured G-MXCP film exhibits excellent mechanical properties, allowing it to be used in a variety of hygroscopic-activation applications. Importantly, the G-MXCP film, with its dense laminar structure, showed no cracks or interlaminar slippage, even after more than 100,000 bending cycles.

**Figure 36:**
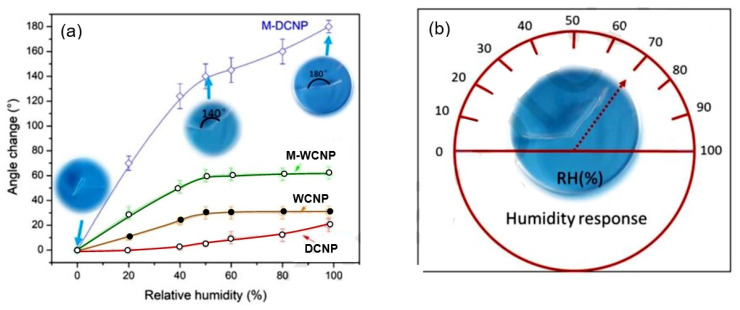
(**a**) Folding-angle changes of tape-like CNP samples under various RH and (**b**) humidity response of M-DCNP sample. The WCNP and DCNP samples were prepared by vacuum filtration using CNFs produced by homogenization in deionized water (WCNFs) and by homogenization in dimethylacetamide (DCNFs), respectively. The DCNP and WCNP samples modified by chitosan (CS) are referred to as M-DCNP and M-WCNP, respectively. Reprinted with permission from [149]. Copyright 2020: Elsevier.

An interesting approach to the development of a humidity sensor was proposed by Wu et al. [149]. They abandoned the use of bilayer films. In their humidity sensor, the authors took films based on cellulose nanopaper (CNP), which was derived, by using various methods, from cellulose nanofibers (CNFs). All the CNP samples were completely double-folded at room temperature and then exposed to humidity. An indicator of the moisture level was the angle between the two sides of the paper. The results of the testing of these devices are shown in Figure 36. The published data indicate that the original shape of the modified CNP after folding immediately responded to water (<3 s) and humidity (<10 min). Similar to other sensors, the shape changes were linked to the swelling effect, which is enhanced in samples modified with chitosan (CS). It was also shown that the introduction of CS on cellulose nanopaper can act as a bonder and strengthening agent to overcome the defects of CNP, thus increasing significantly the dry strength and, in particular, the wet strength, as well as the folding resistance of the final CNP.

**Figure 37:**
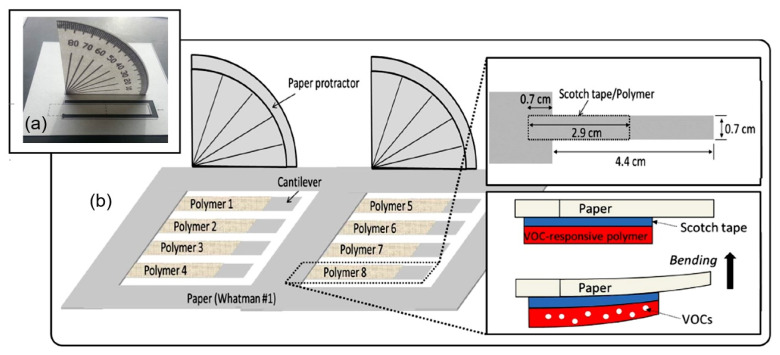
(**a**) Demonstration of paper-based VOC-sensor unit. A protractor and cantilever were patterned on paper. (**b**) Proposed paper-based VOC-sensor array with eight different swellable polymer matrices. Reprinted with permission from [138]. Copyright 2016: Elsevier.

It is worth noting that the bending of sensors that are optimally designed is reversible and directional, and allows cyclicity. In addition, the bending of the cantilever can be controlled by the thickness of the films applied in the sensor. In very thick films, bending is not observed. It is clear that such sensors are not very accurate. However, it is an extremely simple method that allows the monitoring of humidity without external equipment or interface electronics. Thus, with the help of these devices, it is possible to control H_2_O levels with the naked eye. An example of the implementation of these sensors is shown in Figure 37. This device was developed for the detection of volatile organic compounds [138], but can also be used to determine air humidity.

### 7.2. Electroluminescent Devices as PB Humidity Sensors

**Figure 38:**
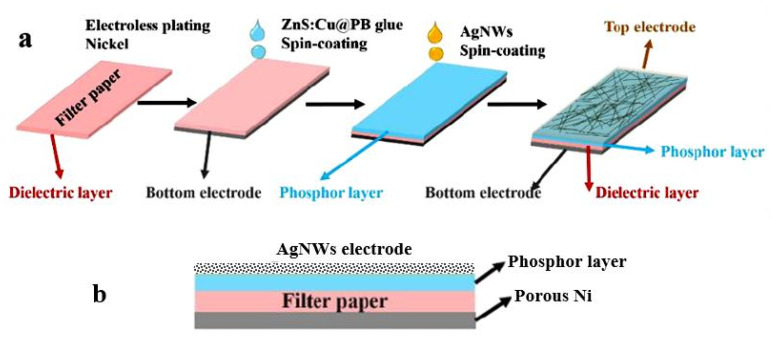
(**a**) Flow chart for the fabrication of paper-based alternating-current electroluminescent devices (ACELs). (**b**) Schematic image of the device. Reprinted from [150]. Published 2019 by MDPI as open access.

Another unusual approach to developing paper-based humidity sensors was proposed by He et al. [150]. They used the effect of humidity on the luminescent properties of ZnS/Cu [151], which is an II–VI compound with unique photoelectronic properties. To manufacture PB humidity sensors, ZnS/Cu phosphor microparticles, with radii of 5–7 μm, were used in several stages (see Figure 38). In the first stage, a thin-film nickel electrode was prepared on one side of the filter paper by using the electroless plating method. Next, a mixture of ZnS:Cu powder and PB glue in a weight ratio of 2:1 was applied to this substrate by using a spin-coating process. The layer was subjected to heat treatment at 120 °C for 30 min. Finally, an Ag NW electrode was applied to the active phosphor layer, followed by annealing at 120 °C for 5 min.

**Figure 39:**
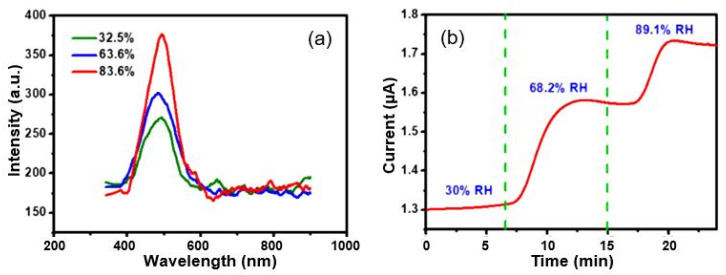
(**a**) The EL spectra of the device under different levels of relative humidity (32.5%, 63.6%, 83.6%) at room temperature. (**b**) Current versus time plot at different (%) RH levels (V = const). Reprinted from [150]. Published 2019 by MDPI as open access.

The functional sensing characteristics of the devices were measured by applying an AC voltage. They are presented in Figure 39. Figure 39b shows the luminescence spectra of an electroluminescent device operating at various humidity levels, while Figure 39a gives the time-dependent properties of the AC current at various levels of relative humidity. All of these results indicate that humidity affects both the luminescent and electrical properties of sensors, and that they can be used as indicators of air humidity. However, He et al. [150] concluded that luminescence intensity is very difficult to calibrate and use in the fabrication of humidity sensors. A change in the glow color could make it possible to visually depict the level of humidity. However, unfortunately, changes in air humidity do not affect the color of the glow, as shown in Figure 39a. Studies have also shown that the number of side effects that can affect the intensity of luminescence is excessive, which significantly limits the use of this method for controlling air humidity. On the other hand, these studies demonstrate that there is potential to develop new humidity-detection technologies that can exploit both the electrical and optical characteristics of materials at the same time.

## 8. PB Humidity Indicators

Moist indicators are not strictly sensors. However, the basis of these indicators is usually paper. Therefore, we decided to include a description of moisture indicators in this review article.

On some occasions, it is desirable to detect only the appearance of moisture in a sample, while the exact concentration is only of secondary importance. This situation can occur during the operation of drying chambers, in which a moisture breakthrough indicates a need for the regeneration of the dryers. In other cases, for example, it is necessary to ensure that the relative humidity does not exceed 30–35%. In many cases, humidity above 30–35% RH is of concern because the corrosion of various materials can occur at this humidity level. Studies have shown that this problem of qualitative and, sometimes, quantitative control over humidity levels can be solved by using cellulose paper impregnated with certain salt crystals that change color when wet. The use of this effect has been found to be the most economical method for detecting changes in humidity levels [152]. Experiments have shown that cobalt chloride is the most suitable material for such applications [153]. The color change is the result of chemical reactions involving cobalt chloride and water. The hydration reaction can be represented by a chemical reaction (1). Two water molecules surround each cobalt atom, forming a dehydrate. Cobalt-chloride dehydrate is purple.

CoCl_2_ (blue) + 2H_2_O → CoCl_2_·2H_2_O (purple) (1)

As the humidity increases further, the crystal structure changes again, this time rearranging to allow four more water molecules to surround each cobalt atom, forming the hexahydrate:

CoCl_2_·2H_2_O (purple) + 4H_2_O → CoCl_2_·6H_2_O (pink) (2)

Heating the hydrated forms of cobalt chloride reverses the above reactions, returning the cobalt chloride to its blue or anhydrous state. Water is “released” in these processes, which are known as dehydration reactions. Thus, this property makes it possible to create a color-changing indicator, which changes from blue to lavender and pink when the humidity increases, and becomes blue again when the humidity reduces. This characteristic has been found to allow these indicators to be reused if they are not exposed to humidity levels higher than 90% RH for 36 h or longer. With these treatments, cobalt chloride is washed out.

Generally, cellulose and cellulose-acetate substrates form the basis of moisture indicators. However, other cobalt-impregnation materials can also be used [154]. Generally, the type of substrate does not affect the color change. It was found that reducing the amount of cobalt chloride in the matrix lowers both the effects of hysteresis and the effective relative humidity range over which the salt/substrate combination can be employed to measure relative humidity. It was also found that the pre-acetylation of cellulose extends the effective range of controlled relative humidity, improves reproducibility, and reduces hysteresis effects [155]. In addition, it is possible to obtain indicators that show a distinctive color change in a narrow range of humidity by optimizing the properties of the substrate, as well as the type and concentration of the cobalt salt [154].

Paper-based moisture indicators are inexpensive, easy to store, and difficult to damage, and they can be used in many fields. The moisture indication can be performed by printing a comparative color guide next to the color-spot indication for ease of reading. Typically, a series of plots is labeled with values of relative humidity, typically in 10% increments from 10% RH to 80% RH, and various combinations of these humidity values.

It has been established that irreversible moisture indicators can be also produced based on cobalt chloride. For these purposes, ammonia salts are added to the element. In the presence of ammonia vapor, the elements lose their color reversibility. These irreversible elements, frequently referred to as maximum-humidity-indicator cards, are necessary, for instance, to show when certain levels of humidity are exceeded or the recharging/replacement of a desiccant. These indicators can mark the highest humidity level encountered by cargo during a voyage regardless of current (potentially lower) humidity levels. Maximum moisture indicator cards provide a clear and unmistakable means of determining whether goods are exposed to harmful levels of humidity during their journey.

As stated above, cobalt salts are water-soluble and, therefore, should not be in direct contact with water or steam, nor should they be exposed to extremely high humidity for extended periods of time. If these indicators are stored in a humid environment, they may become inaccurate. They are damaged by excessive moisture or condensation, which dilutes cobalt salt and results in nonuniform chemical distribution and inconsistent color response [156]. Strong solvents, such as ammonia vapors, which cause the discoloration of the color-changing dye and lead to color irreversibility, should not be present in the atmosphere.

It should be noted that other salts can be used in humidity indicators, in addition to cobalt salts. Their development primarily followed a directive issued by the European Community (EC), in 1998, which classified products containing cobalt (II) chloride from 0.01 wt.% to 1 wt.% as toxic. Studies have shown that there is a fairly large range of materials that can also change color when exposed to a moisture [157,158,159,160,161,162,163,164,165,166,167]. For example, Matushima et al. [159,160,161] presented a series of reports on reversible humidity-sensitive indicator films prepared from the combination of a dye with a sugar-based hydrogel, e.g., agarose or k-carrageenan, that undergo reversible color changes due to the appearance of an aggregated form of the dye at high humidity and its subsequent disintegration at low humidity. In these humidity indicators, the color change is due to the aggregation of the dye caused by humidity. In 2010, Mills et al. [165,166] published the details of a humidity indicator based on thiazine dye encapsulated in a urea-containing hydroxyethyl cellulose (HEC) film. In this system, urea was present at about 20 times the amount of dye. Under both dry conditions (i.e., RH = 0%) and ambient conditions (RH~60%, at 23 °C), the film was bright purple, but it turned dark blue after exposure to ambient atmosphere with a relative humidity of ≥85%. Copper(II) chloride and potassium–lead iodide can also be used in humidity indicators. Yellow lead iodide is obtained from potassium iodide and lead is obtained through the adsorption of moisture. The experiment showed that polymer–dye systems also change their color when the air humidity changes [168].

**Figure 40:**
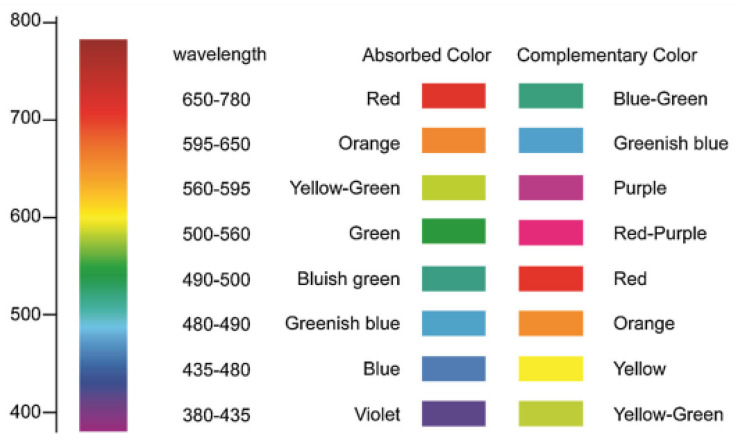
Absorbed and complementary colors of white light. Reprinted with permission from [169]. Copyright 2018: Springer.

Research has shown that humidity indicators can be developed on other principles. In particular, numerous studies have been carried out on cellulose nanocrystals (CNC), with the aim of studying reflection-color changes under the influence of air humidity. Table 13 shows the results of these studies, and Figure 40 shows the correspondence between the color and the wavelength of the white light. In these studies, it was found that the effect of moisture on the colors of the films was repeatable and reversible. This effect is based on the swelling of NCs in a humid atmosphere, which causes an increase in the thickness of the sample, accompanied by a change in the color of the film.

**Figure 41:**
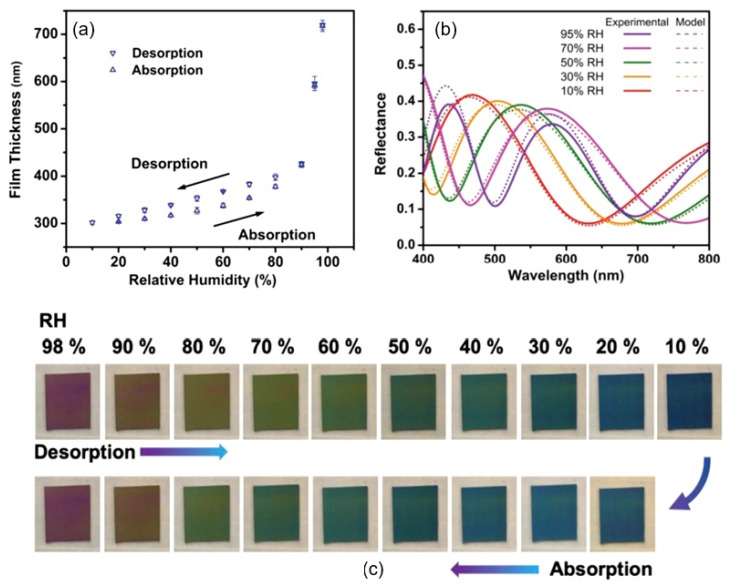
The correlations between film thickness, color, and relative humidity. (**a**) Thickness of a (CHI/CMC-N_3_)_25_ film at different humidity levels. (**b**) Comparison between experimental and model reflectance spectra of a (CHI/CMC-N_3_)_25_ film during desorption process. (**c**) Digital images of a (CHI/CMC-N_3_)_25_ film at different humidity levels. Reprinted with permission from [181]. Copyright 2019: Wiley.

As can be seen from Table 13, both pristine CNC and the composites based on it can be used to manufacture moisture indicators. For example, Dong et al. [181] proposed the use of a sandwiched a cellulose-derived double-network hydrogel between two layers of gold for this purpose. This sandwich structure was composed of chitosan (CHI) and a photoreactive derivative of carboxymethyl cellulose–azido (CMC-N_3_). This combination of polyelectrolytes, CHI and CMC, was chosen as the building material because CHI and CMC contain amino groups and carboxyl groups, respectively, which are charged in aqueous solutions and are easily chemically modified. These CHI/CMC-N_3_ films can swell/de-swell at various humidity levels. Due to the excellent hygroscopic swelling properties of cellulose, the thickness of the cellulose-derived hydrogel layer enhanced with increasing ambient humidity (Figure 41a). This led to the redshift of its reflection spectrum (Figure 41b). In particular, the Au–cellulose–Au structure had an orange-red color when completely dry, while it turned green in the semi-swelled state and became bright red in its fully swollen state (Figure 41c). Dong et al. [181] state that this humidity-monitoring method is inexpensive and easy to prepare, and that it is highly scalable and durable.

In addition to the chitosan, other components were used in CNC-based composites, in order to improve the parameters of CNC films. For example, the addition of sodium-chloride electrolytes shielded the negative charge of the sulfate groups on the surfaces of CNCs and reduced the chiral nematic distance of the CNCs, resulting in improved selectivity in the CNC films at high humidity levels [178]. Poly(ethylene glycol) (PEG) can be used as a plasticizer to improve the mechanical strength and moisture sensitivity of CNC films [169,170]. For the same purpose, glycerol, a common small-molecule polyol, was introduced into the composite, which is also a plasticizer [172,173,174]. In addition, the strong water-absorption property of glycerin improved the water-absorption capacity of the CNC film and slowed the water-evaporation rate in the evaporation-induced self-assembly process. By changing the ratio of materials in the composite, it is also possible to control the color range of the indicator. For example, increasing the concentration of glycerol in the CNC/glycerol composite within 0–40 wt.% shifted the reflection peak from 280 nm to 620 nm; the color of the film changed from blue to red. Zhang et al. [179] applied N-methylmorpholine-N-oxide (NMMO) to adjust the CNC-helix length and increase the adsorption/desorption capacity of water molecules on CNC films. The addition of highly hydrophilic polyacrylamide (PAM) further enhanced the swelling effect, resulting in a redshift at the reflectance peak of the composite, showing a more pronounced color change [175]. Hydrazone modified poly(nisopropylacrylamide) (PNIPAM) with temperature-sensitivity properties was assembled by CNC to obtain an indicator that was sensitive to both temperature and humidity [176,177].

However, it must be borne in mind that the application of composites requires careful control of the composition and preparation conditions. For example, excess NaCl destroys CNC alignment. Grafting PNIPAM onto CNC surfaces by using chemical bonding reduces the toughness of CNC films [177]. The addition of PAM reduces the stability of the indicator readings during repeated use. Thus, the redshift of the reflectance spectra of the CNC/PAM composite decreased markedly after 10 cycles [175]. If the redshift in the first cycle was from 580 nm to 760 nm, then the peak was at 720 nm after 10 cycles.

## 9. Challenges in Paper-Based Humidity Sensors and Trends in Development

**Figure 42:**
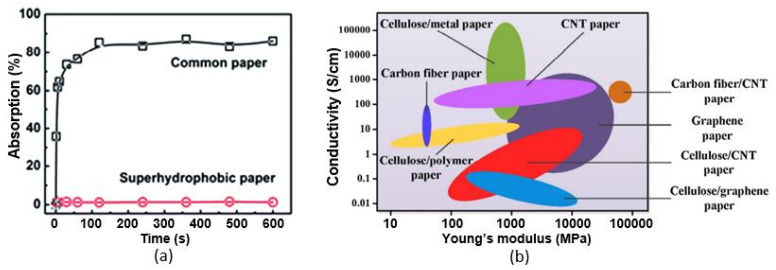
(**a**) Plots of water-absorption versus soaking time of conventional (common) and superhydrophobic paper. Hydrophobic properties were imparted to common printing papers by applying superhydrophobic coatings based on a block copolymer of polystyrenepoly(methyl methacrylate) (PS-co-PMMA) and SiO_2_ NPs. Reprinted with permission from [189]. Copyright 2014: Royal Society of Chemistry. (**b**) Material-selection diagram: electrical conductivity plotted against Young’s modulus. Young’s modulus, as a symbol of the mechanical strength, and electrical conductivity, representing the electrically conductive property, are compared across a variety of paper materials. The diagram is helpful in providing design guidelines for the selection of substrate materials for the fabrication of paper-based devices. Reprinted with permission from [192]. Copyright 2017: Elsevier.

**Figure 43:**
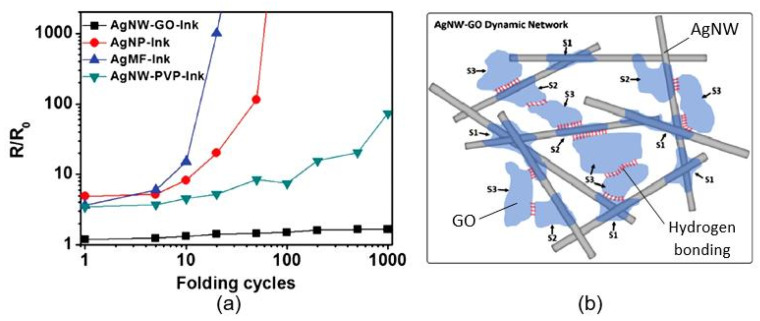
(**a**) Relative changes in resistance of AgNW-GO-, AgNW–PVP-, AgNP-, and AgMF-based electrodes drawn on paper substrates during 1000 repeated folding–unfolding cycles. (**b**) Schematic diagram illustrating the structure of the AgNW–GO-ink electrodes. Reprinted with permission from [210]. Copyright 2017: Wiley.

As shown in this review, the developers of paper-based humidity sensors have made significant progress over the past decade. However, numerous challenges remain and PB humidity sensors are still far from reaching the sensor market [37,44,182,183,184]:

(1) PB humidity sensors usually have a fairly high sensitivity, with RH > 40% range. Sensors capable of operating in this sensitivity range are suitable for most applications. However, for special applications, it is desirable for sensors to operate effectively in the 10–40% RH range. Another limiting factor of most PB humidity sensors is their slow response and recovery. Consequently, PB humidity sensors cannot completely return to their original state within such certain time intervals (≈3–5 s) at room temperature, which reduces the accuracy of their measurements during cyclic changes in humidity [185]. Therefore, developments aimed at improving the parameters of PB humidity sensors, such as their sensitivity to low humidity levels and their speed of response and recovery, will undoubtedly contribute to the expansion of the range of possible applications for these sensors. Based on the research results presented above, in order to solve this problem, reducing the distance between the electrodes, optimizing the structure, and reduce the thickness of the humidity-sensitive layer are proposed.

(2) Paper is sensitive to the state of the surrounding atmosphere, so PB devices require harsh storage conditions. Failure to comply with these conditions significantly reduces the period of storage and operation [186]. This also limits the commercial application of PB sensors, which is an important disadvantage. It means that addressing the problem of convenient and long-term storage is crucial for promoting the widespread commercial use of paper-based sensors in the future. It is hoped that this problem can be solved by using special grades of paper and surface functionalization.

(3) Various applications have specific paper requirements. Therefore, the ability to use paper with distinctive properties to meet the requirements of individual applications can be an effective approach to optimizing the performance of PB humidity sensors. For example, the use of hydrophobic paper makes it possible to reduce the influence of the paper substrate on the characteristics of humidity sensors (see Figure 42a) when using polymeric and solid-state humidity-sensitive materials. Highly conductive paper substrates can be used as electrodes in these sensors. Fire-retardant paper increases fire safety. Transparent papers are of particular interest as platforms for the research into and development of optical humidity sensors. However, to realize these possibilities, technologies that can reproducibly produce papers with the desired properties are required. Currently, numerous studies on this topic are underway [187,188,189,190,191,192], but they are still in their infancy. Some of the results of these studies are shown in Figure 42b. Liana et al. [193] believe that if paper is increasingly recognized as a substrate material suitable for the development of various sensors and electronic devices, there will be competition between manufacturers for paper types with additional functionality.

(4) It is necessary to adapt the technology used to manufacture paper-based humidity sensors as much as possible to the requirements of mass production [194]. In addition, the fabrication of most flexible paper-based humidity sensors is based on methods with which it is relatively difficult to ensure reproducibility and device stability from across batches. Only simple but reproducible and high-performance technologies will facilitate the successful commercialization of paper-based humidity sensors [184].

(5) The technologies used to manufacture flexible electrodes on paper substrates require further improvement. The electrodes used in PB humidity sensors should generally meet the following requirements: (i) good electrical conductivity; (ii) excellent mechanical flexibility; (iii) good adhesion to paper; (iv) a manufacturing process involving technologies compatible with mass-scale production; (v) low cost; and (vi) non-toxicity/low toxicity. Unfortunately, many of these requirements for electrodes in PB sensors are not met. The development of printable conductive inks and the use of advanced printing technologies [195] for the manufacturing of PB flexible electrodes are effective ways of overcoming the shortcomings described above. For example, Duan et al. [196] believe that the use of daily carbon ink (DCI) may be a promising approach to solving this problem. According to Duan et al. [196], DCI can be applied in humidity sensors due to its many unique characteristics and advantages, such as good conductivity and dispersion, strong adhesion, black color, and low cost.

(6) Further challenges include the durability and stretchability of paper. The stretching properties of paper are generally poor. Therefore, great efforts should be undertake to conduct studies on this topic [197,198,199,200], as well as on the optimization of the surface and mechanical properties of paper materials themselves, which are closely related to the performance of PB humidity sensors. The surface properties of paper directly affect the adsorption of H_2_O molecules and the parameters of both humidity-sensitive materials and the electrodes formed on their surfaces. Furthermore, the stability of the parameters of bent sensors depends on the mechanical flexibility of the paper used. Stress–strain dependencies are crucial for studying the flexibility of materials, but they can only be determined for solid materials, while the structure of paper is characterized by heterogeneity and randomness [201]. In addition, the modulus of elasticity of paper depends on its moisture content. Higher moisture content softens the material, thereby lowering the modulus of elasticity of paper [202].

(7) When developing PB humidity sensors, various humidity-sensitive materials can be used. However, they do not always determine the characteristics of humidity sensors, especially those of the resistive type. Very often, the resistance of humidity-sensitive materials is either excessively low resistance or excessively high. In the first case, the range of resistance changes is narrow, and in the second, the sensory response is controlled not by a sensitive material, but by a paper substrate. Neither case is optimal for achieving the best performance of sensors that use additional humidity-sensitive materials. Therefore, technologies for the synthesis and formation of humidity-sensitive layers with the required electrical parameters, which are optimal for use in humidity sensors, are urgently required. Paper-surface-passivation technologies are also necessary to reduce the influence of paper on the parameters of PB humidity sensors with additional humidity-sensitive materials.

(8) New sensitive materials for PB humidity sensors need to be explored. At present, carbon and its derivatives (e.g., CNTs, graphite, graphene, GO, rGO, and carbon black) are the main sensing materials for PB humidity sensors. It is expected that the use of new materials will improve the performances of these sensors [14]. Further research may focus on exploring new sensing mechanisms and finding innovative nanomaterials to address the observed instability of PB humidity sensors [14,184]. It is believed that promising recently developed nanomaterials, such as quasi-two-dimensional (2D) materials, including black phosphorus (BP) [203], boron nitride (hBN), transition-metal dichalcogenides (TMDC) [204], carbonitrides, and nitrides (MXenes) [205,206,207], could be employed. However, for the application of high-quality 2D film materials to rough and porous paper-substrate surfaces, many challenges need to be solved by optimizing paper-surface characteristics and developing new material-preparation methods [183].

(9) The impact of mechanical flexibility on PB humidity sensors must be reduced [183,208]. Paper has good flexibility, but mechanical bending/distortion has a significant impact on the performance of flexible PB humidity sensors. For example, strong bending can damage the sensing layer and electrical contacts [40,209]. In particular, it has been found that tensile stress in silver-ink films causes deformation at the point of bending, which leads to increased resistance. Figure 43 shows the effect of multiple foldings on the resistance of Ag-based electrodes fabricated from various inks.

Based on the results obtained by Liu et al. [210], it was concluded that the poor foldability of AgNP- and AgMF-ink-based electrodes on paper substrates was mainly due to (1) the relatively rigid structure of the electrodes, consisting of Ag nanoparticles (NP) and Ag microflakes, and (2) the relatively poor adhesion of the electrode material to the cellulose paper. Regarding the AgNW–PVP-ink electrodes on paper, the resulting breaks did not grow into larger defects after 1000 folding cycles, although a number of mechanical defects appeared on the electrodes after only one cycle of folding. It is assumed that AgNW sliding in the AGNW–PVP-ink conducting network due to the AgNW structure helped to compensate for part of the strain attached to the electrodes, which gave the electrodes better flexibility compared to the electrodes based on the AgNP-ink and AgMF-ink [211].

Regarding the AgNW-GO-ink electrodes, it is believed that their flexibility was mainly associated with two factors (Figure 43b). Firstly, the GO sheets, which wrapped and connected with the AgNW (S1, S2), significantly improved the mechanical strength of the AgNW network. Secondly, the oxygen-containing functional groups on the GO surface contributed to the better adhesion between the AgNW–GO-ink electrodes and the surface of the cellulose through hydrogen bonding [212]. These results were consistent with the conclusions of other researchers, which indicated that the stability of flexible PB sensors can be improved by using nanostructured materials, such as Ag nanofibers and nanowires [53,213]. It was found that these materials are very resistant to deformation, and their causes almost no lateral cracks.

(10) Despite the great importance of multifunctional PB sensors, their development faces significant challenges [28,184,214,215,216]. Although their large area and customizable/printable paper characteristics make the manufacturing of multi-functional integrated PB sensors on single sheets of paper simple, multi-functional integrated PB sensors still involve cross-sensitivity to various environmental stimuli. According to Tai et al. [183], this problem can be solved through several approaches, which are described below.

Multifunctional PB sensors can be obtained by searching for materials specifically designed to detect gas, humidity, temperature, and strain [217,218,219,220,221]. These materials can be deposited in spatially resolved areas of the paper substrate and integrated through appropriate packaging strategies.

According to the perceptual characteristics of different stimuli, cross-sensitivity effects can be reduced by means of various signal-conditioning modes, e.g., resistance, capacitance, frequency, and current.

Multifunctional BP sensors can be implemented by combining a number of data-processing techniques, e.g., pattern recognition, principal component analysis, and machine learning.

**Figure 44:**
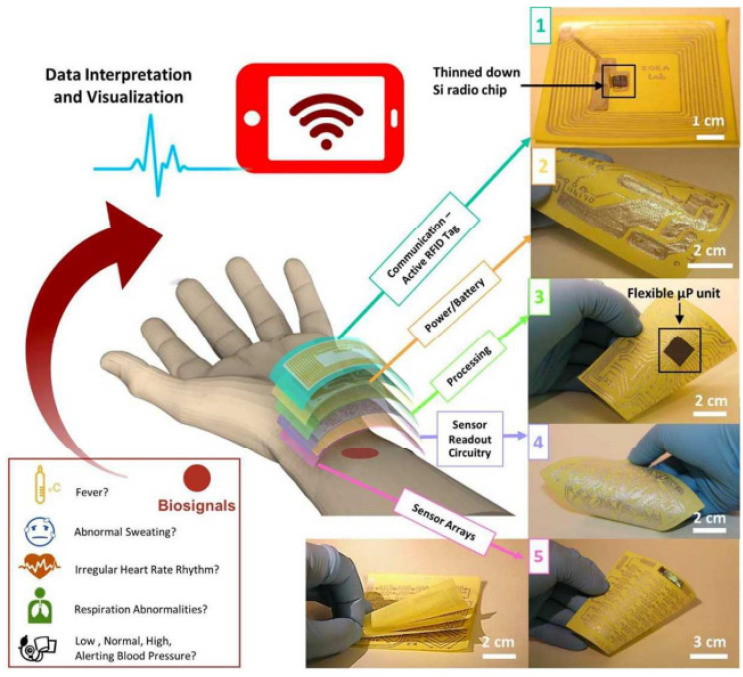
Three-dimensionally stacked paper-based healthcare-monitoring system. Beginning from the top, layer (1) contains an active RFID tag printed on paper with a flexible Si-based radio chip for wireless data communication; layer (2) contains a printed power source; layer (3) contains the processing unit with a flexible Si-based microprocessor (µP); layer (4) contains sensor readout circuitry fully printed on paper; and layer (5) contains the sensor arrays. Reprinted with permission from [208]. Copyright 2017: Wiley.

An example of a multifunctional paper-based testing system, developed for a healthcare monitoring [208], is provided in Figure 44. The system was implemented as a hybrid design on cellulose nanopaper using AgNP-based inks for interconnects and thin Si-based chips for active circuit components. The data collected from the sensors were wirelessly transmitted to a smartphone and visualized in real time. Examples such as this show that nanopaper can be a suitable platform for wearable healthcare-monitoring systems and multi-functional sensors [222].

**Figure 45:**
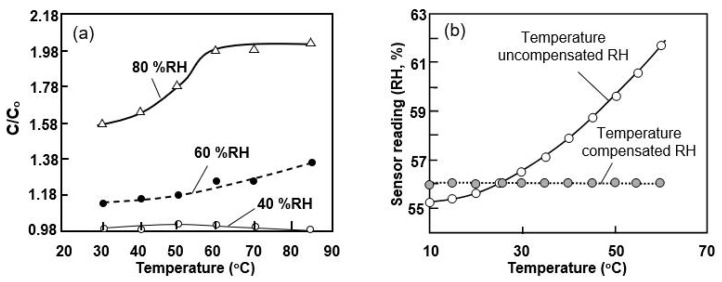
(**a**) Temperature influence on the capacitance of the inkjet-printed paper-based sensor at different humidity rates, for a fixed frequency of 100 kHz. Lumi silk (90 g/m^2^) from Stora Enso (Helsinki, Finland) was used as substrate material and Ag NPs were used for interdigitated electrodes. Reprinted from [22]. Published 2017 by MDPI as open access. (**b**) The effect of temperature on the readings of a capacitive humidity sensor based on aluminum oxide with and without compensation for temperature changes using the ANN method. Reprinted from [223]. Published 2015 by IFSA Publishing, S.L., as open access.

(11) Notably, such processes as the adsorption and desorption of H_2_O molecules, as well as ionic diffusion in humidity-sensitive materials, are dependent on temperature. As the temperature rises, the conductivity of the materials used in the sensor increases in turn. Furthermore, the absolute humidity content, calculated as ordinary values in ppm (particles per million), increases as the temperature rises at a certain RH. This means that the characteristics of humidity sensors are also turn affected by the ambient temperature [223,224], which can vary widely. An example of the influence of temperature on the readings of a capacitive humidity sensor is shown in Figure 45. it can be observed that the variation in the ambient temperature leads to significant errors in the determination of the humidity. Subsequently, the effect of the temperature increases with higher humidity levels (Figure 45a). For lower humidity rates (around 40 RH %), the sensors show low sensitivity to temperature, while above this humidity level, the sensitivity becomes more dramatic.

**Figure 46:**
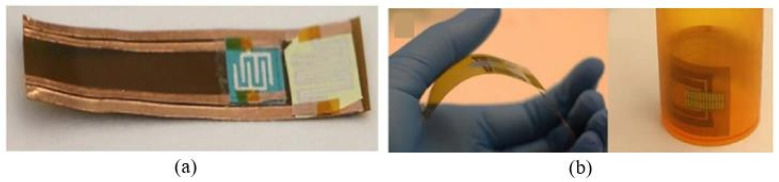
(**a**) Sticker containing paper-based temperature and humidity sensor. Reprinted with permission from [231]. Copyright 2021: ACS. (**b**) Flexible electronic interface decal attached to a paper sensor decal in bent form, displayed in a hand (left photograph) and inside a prescription bottle (right photograph). Reprinted with permission from [232]. Copyright 2019: IOP.

Two solutions to this problem are possible. One (i) is to stabilize the temperature of the sensor, which is an energy-consuming method and increases the size of the measuring device. The other (ii) is to control the temperature, alongside the measurement of the humidity, and to automatically make appropriate corrections to the sensor readings [223,225,226,227]. The second option seems to be more rational, especially if this task is combined with the development of multifunctional integrated PB sensors. Furthermore, Islam et al. [228] believe that temperature compensation can be performed better by using software methods that provide greater flexibility in terms of humidity sensors. In particular, these authors suggested applying artificial neural networks (ANN) in cases such as this. Artificial neural networks have emerged as highly effective learning techniques that are suitable for performing nonlinear, complex, and dynamic tasks with a high degree of accuracy [229]. However, compensation with the hardware method has low accuracy and high cost. It is important to note that combinations of PB humidity and temperature sensors have already been developed and, have even been successfully used in commercial devices. For instance, Khan et al. [230,231] demonstrated the use of paper-based temperature and humidity sensors in the healthcare industry, particularly when placed inside a prescription container (Figure 46b).

**Figure 47:**
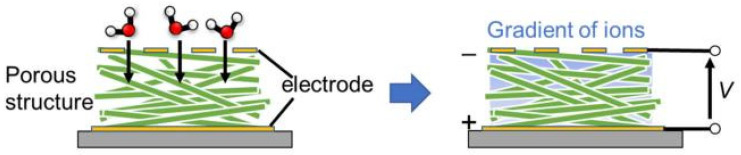
Typical mechanism of voltage generation from ambient humidity. A porous nanowire network charged in negative, separately charged ions. Thus, a charge imbalance is formed and a potential difference is generated by humidity. Reprinted from [13]. Published 2022 by MDPI as open access.

(12) It is necessary to consider the development of integrated and automated systems based on paper platforms with multiple functions, such as sample preparation, separation, reaction, detection, processing, and the transmission of information [233]. For real analyses, it is necessary to design a more reproducible electronic system in combination with a portable and convenient reader. Currently, a new generation of flexible optoelectronic, electronic, and sensory devices on cellulose substrates is emerging [234,235,236,237,238,239,240,241,242,243,244], which opens up promising opportunities for research on and the development of low-cost integrated systems.

(13) The final issue is the ability to develop self-powered humidity sensors for wearable electronics [185,236,245,246]. Various research groups are currently working on this topic. It was shown that paper is a versatile substrate for creating various energy-generation and -storage devices, which can be used in self-powered nanosystems [13,236,247,248]. The most interesting approach is the use of a battery-less system, in which power is generated by the humidity of the environment. This approach was discussed in [249,250,251,252] and is based on conducting metal–air redox reactions, which are commonly used in high-performance metal–air batteries. These batteries can convert chemical energy into electrical energy and do not require external stimulation, demonstrating the advantage of achieving high stability. The self-powered chemoelectric device consists of a multilayer sandwiched structure with metal electrodes and solid electrolytes. When the device is exposed to humid air, the water concentration gradually changes in the solid electrolyte. On the top side, the concentration of adsorbed water is higher than on the bottom. This induces a diffusion of ionic species related to water molecules (protons and hydronium ions), which subsequently generates a potential difference, as shown in Figure 47. Depending on the components used, a moisture-enabled electricity generator (MEEG) can provide an open-circuit voltage of 0.1–0.5 V.

**Figure 48:**
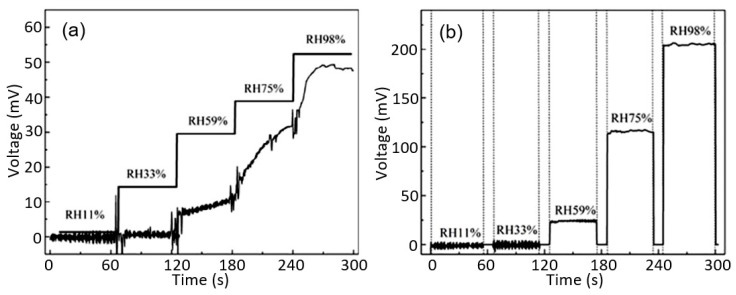
Open-circuit-voltage response of CoCl2@FP humidity sensor. (**a**) Open-circuit voltages in response to increases in RH to 11, 33, 59, 75, and 98% for 1 min. (**b**) Open-circuit-voltage-stability response intercepted for 1 min in RH of 11, 33, 59, 75, and 98%. Reprinted with permission from [253]. Copyright 2021: Elsevier.

Examples of the implementation of self-powered chemoelectric PB humidity sensors were presented in works by Kan et al. [253] and Li et al. [254]. Kan et al. [253] used cellulose filter paper (FP) in the process of manufacturing humidity sensors. The FP was completely immersed in CoCl_2_ solution and maintained for 6 h, so that the cobalt-chloride solution could fully penetrate into its pores. A conductive tape was used as electrodes, equipped with a hole for penetrating water vapors. Figure 48a illustrates the response of the sensor to the changes in humidity. The device provided a certain open-circuit voltage response in the overall humidity range; if the humidity was higher, the voltage response was larger. When the RH was below 59%, the open-circuit voltage slowly increased. When the RH exceeded 59%, the voltage increased sharply, and the response curve became relatively. To determine the maximum output voltage of the sensor at different humidity levels, the CoCl_2_@FP sensor was placed in each humidity environment for 10 min to obtain the open-circuit voltage in the stationary state. At low humidity levels, of 11–33% RH, the voltage of the sensor approached 0 V (Figure 48b). When the RH increased to 98%, the open-circuit voltage rose to over 200 mV. The response and recovery times of the CoCl_2_@FP sensor for electric humidity sensors are long (143 s) and short (45 s), respectively. Kan et al. [253] stated that the voltage response of the sensor showed no significant differences, even after long times and repeated tests, which indicated the excellent stability performance of the sensor. In addition, this device can maintain excellent voltage-response performance at operating temperatures of 0–40 °C and after 3000 folds. Li et al. [254] found that polypyrrole (PPy), which has high electrical conductivity, good environmental stability, and reversible electrochemical redox characteristics, can also be used to produce self-powered chemoelectric humidity sensors. In their study, a humidity sensor was prepared using the in situ polymerization of PPy in filter paper doped with acetic acid. The results demonstrated that the PPy/filter-paper-based humidity sensor provided an electron-migration channel with the ability to detect the relative humidity. External humidity stimulation can regulate the electrochemical reaction of PPy. The PPy system loses electrons to form carrier directional channels, resulting in changes in the output voltage measured between the two electrodes. According to Li et al. [254], the PPy/filter-paper-based humidity sensor exhibited moisture-dependent voltage responses over a wide range of relative humidity, of 11–98%, and a response/recovery time of 43/51 s.

**Figure 49:**
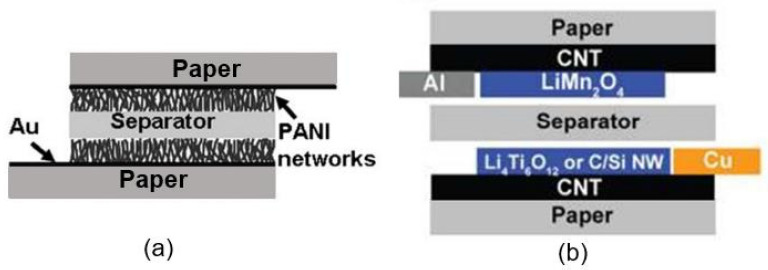
(**a**) The configuration of PB supercapacitors developed by Yuan et al. [259]. Reprinted with permission from [259]. Copyright 2012: Wiley. (**b**) The use of CNT nanopaper as a collector for LiMn_2_O_4_ nanorod cathode and Li_4_Ti_5_O_12_ nanopowder anode in Li-ion battery. Reprinted from [266]. Published 2009 by National Academy of Sciences as open access.

Another solution is to drive a fast-response PB humidity sensor with another powered system. For example, microbial fuel cells (MFCs) [255], fluidic batteries [256], flexible thermoelectric nanogenerators (TEGs) [257], and supercapacitors (SC) [214,237,258,259] can be produced from paper for this purpose. In particular, Yuan et al. [259] successfully fabricated all-solid flexible polyaniline (PANI) supercapacitors on paper substrates to store sufficient energy generated by a piezoelectric generator or solar cell to power various sensors (see Figure 49). He et al. [260] developed a triboelectric nanogenerator based on cellulose fibers. In addition, several scientific groups are working to improve the efficiency of power generation using moisture [261,262]. All these observations suggest the possibility of developing self-powered paper-based humidity sensors [263,264,265].

## 10. Summary

While paper offers significant potential as a development platform for humidity sensors, further improvements in fabrication and analysis methods are still needed to match the performance of the conventional humidity sensors currently on the market. The development of paper-based sensors is still in its infancy; therefore, currently, limitations remain regarding the sensitivity, accuracy, repeatability, and stability of paper-based sensors.

After reviewing the parameters of paper-based humidity sensors, it is clear that extensive developments have taken place in recent years that have significantly improved the performances of these sensors, and there is no doubt that further advances will be made in this growing field in the future. This means that adequate sensor properties (high sensitivity, good selectivity, stability, linearity, and acceptable response and recovery times) will be achieved, and that these sensors will be used in the same areas as sensors on solid substrates and flexible polymer substrates. However, this requires further research related to the improvement of conventional methods and the search for new methods for the deposition of functional humidity-sensitive and electrode materials on paper surfaces, in order to overcome the concerns described in this study and to design better and more stable devices. Further in-depth and detailed research on paper-based sensors will also play a positive role in promoting their future maturity and commercialization. However, of course, when solving these problems, the simplicity and cost advantages of paper-based sensors must not be sacrificed [193].

## Figures and Tables

**Table 1:** Summary of paper-based capacitance humidity sensors with paper sensing elements.

Sensing Material	Electrode	RH Range (%)	Response	Res/Rec Time	Ref.
Recycled paper, cardboard, food-packaging paper	Ag, screen printing	35–80	~3–6 (C/C_0_)	N/A	[17]
Paper (Lumi silk)	Ag, inkjet printing	40–100	∼3.5 (C/C_0_); 2 pF/% RH (ΔC/ΔRH)	4/3 min	[22]
Cellulose paper	Pencil drawing	40–90	~2 (C/C_0_) 4.5%/% RH (ΔC/ΔRH)	2/7 s	[25]
Cellulose paper	Pencil drawing	31–90	∼2.2 (C/C_0_); 0.06 pF/% RH	10/32 s	[26]
Tissue paper	Copper tape	40–95	~4 (C/C_0_); 11 pF/% RH	1.5/2.2 s	[21]
Functionalized copy paper	Ag, gravure printings	30–90	~15 (C/C_0_);	~1/~1 s	[27]
Post-it-note paper	Ag ink pen	10–95	0.18%/% RH	1.2/2.3 s	[28]
Functionalized p_e:smart paper type 2	Ag, inkjet printing	20–80	~10 pF/% RH ~10 (C/C_0_)	Long response	[16]
Metallized papers	Al, laser ablation	2–60	0.1 pF/% RH ~1.5 (C/C0)	260/20 s	[18]
		60–85	6 pF/% RH ~15 (C/C0)		
Glossy paper	Graphene, inject printing	10–70	1.8 (C/C_0_) 0.03 pF/% RH (ΔC/ΔRH)	N/A	[29]

**Table 2:** Summary of paper-based capacitance humidity sensors.

Sensing Material	Method	RH Range (%)	Response	Res/Rec Time	Ref.
CNT (10%)—paper composite	CNT-cellulose suspensions were filtered, pressed, and dried to form hand sheets	40–80	22% (ΔC/C_0_); ~9 fF/% RH (ΔC/ΔRH)	Slow	[30]
MWCNT/PDMS (photo paper)	Screen printing	70 70–95	0.375 pF/% RH 8.24 pF/% RH	N/A	[31]
GO	Soaking	30–90	209% (ΔC/C_0_); 5.65 fF/% RH (ΔC/ΔRH)	Slow recovery	[32]
GO	Printing	30–90	85–320 (C/C_0_)	170/40 s	[33]
GO/CNC (on PET)	Drop coating	25–90	~2·10^3^ (C/C_0_)	17/22 s	[34]
Aluminum oxide	Electrochemical oxidation	50–80	4.5 pF/% RH (ΔC/ΔRH)	Slow	[19]
Porous silicon	Spray-coating	60–95	>4.5 (C/C_0_)	>50/20 min	[35]

CNC—cellulose nanocrystal.

**Table 3:** Summary of conductometric (resistive) full-paper humidity sensors.

Type of Paper	Electrode/Method	RH Range (%)	Response	Res./Rec. Time	Ref
Printing paper	Tape pasting	41–72 72–91.5	~8 (I/I_0_) ~200 (I/I_0_)	472/19 s	[37]
Printing paper		58–75 75–99	5.45 kΩ/% RH (ΔR/ΔRH) 411 Ω/% RH.	382/22 s 991/48 s	[38]
Tracing paper	Graphite, line-patterning	20–70	215% (ΔV/V)	N/A	[39]
Whatman 3MM Chr	Graphite, ball-point pen	30–90	N/A	1500/- s	[40]
Printing paper	Au, sputtering	20–70	14 (R_0_/R)	N/A	[41]
Glossy photo paper			12.5 (R_0_/R)		
Glossy photo paper	Ag, inkjet printing	20–90	~10^4^ (R_0_/R)	N/A	[42]
Glossy photo paper	Ag, PEDOT:PSS inkjet printing;	5–85	~3 × 10^4^ (R_0_/R)	~10/~5 s	[43]
HP advanced photo paper	Ag, inkjet printing	30–60 60–90	~3 (R_0_/R) 7 × 10^3^ (R_0_/R)	N/A	[18]
Printing paper	Carbon, spray	20–90	~800 (I/I_0_)	237/29 s	[44]
TEMPO-oxidized paper	Laser irradiation	11–98	1.2 × 10^3^ (R_0_/R)	60/495 s	[45]
Conventional cellulose A4 paper	Ag, inject printing	15–92	~1.25 (R_0_/R) 0.57 kΩ/% RH	294/306 s	[46]
Cellulose/KOH composite	N/A	11–98	~2 × 10^2^ (R_0_/R)	6/11 s	[47]

**Table 4:** Comparison of the performances of humidity sensors based on cellulose- and carbon-based materials.

Material	Deposition	Electrode/Method	Range % RH	Response	Res./Rec. Time	Ref.
CNT/cellulose	Rod-wire coater	Silver	10–95	∼70% (−ΔI/I_0_)	321/435 s	[54]
TEMPO-oxidized cellulose fibers/functionalized CNTs paper	Vacuum filtering	Silver	11–95	∼87% (−ΔI/I_0_)	333/523 s	[55]
Nanofibrillated cellulose/MWCNT composite film	Vacuum filtration	Copper-foil tape	11–95	∼70% (−ΔI/I_0_)	330/377 s	[56]
Regenerated cellulose/CNT composite fiber	Wet spinning	N/A	35–86	44% (ΔR/R_0_)	N/A	[57]
Cellulose/CNT composite film		N/A	35–85	~60% (ΔR/R_0_)	N/A	[58]
Functionalized MWCNT-coated paper	Spray coating	Nano-metal inkjet printing	20–90	∼60% (−ΔG/G_0_)	N/A	[59]
H_2_SO_4_ and HNO_3_ (3:1)-treated o-MWCNT-coated paper	Inkjet printing	Graphite, pencil, hand-drawing	33–95	∼30% (−ΔI/I_0_)	470/500 s	[51]
COOH-functionalized SWCNT-coated paper	Filtration	Copper-foil tape	10–75	37.5% (ΔS/S_0_)	6/200 s	[60]
Graphene nanoplatelet	Vacuum filtration	Copper	5–90	290% (ΔR/R_0_)	12/20 s	[61]
	Dip coating			75% (ΔR/R_0_)	9/15 s	
CNF/GNP composite on PEN	Screen printing	Ag, screen printing; GNP/CNF	30–90	∼140–240% (ΔR/R_0_)	17/22 s	[62]
Graphene–carbon (glossy photo paper)	Screen printing	Graphene–carbon	30–92	13 Ω/% RH	4/6 s	[63]
Graphite	Screen printing	Ag, inject printing, laser ablation	N/A	0.0564%	N/A	[64]
Graphite (hard cellulose paper (business card))	Pencil drawing	Graphite	43–83	1.4–8.6 kΩ/% RH 0.5–2.5%/RH	270/420 s	[65]

CNT: carbon nanotube; GO: graphene oxide; rGO: reduced graphene oxide; CNC: cellulose nanocrystal; Gr: graphene; GNP: graphene nanoplatelets; CNF: cellulose nanofiber.

**Table 5:** Summary of conductometric (resistive) paper-based humidity sensors.

Sensing Material	Method	Range RH (%)	Response	Res./Rec. Time	Ref.
SiO_2_/acetate film	Hand drawing	50–80	~10^4^ (R_0_/R)	31/7 s	[72]
CdS NPs	Drop casting	5–99	55% (ΔR/R) ~2 (R_0_/R)	~75/~50 s	[73]
ZnO NPs	Spin coating	20–70	14 (R_0_/R)	600/- s	[41,74]

**Table 6:** Summary of conductometric (resistive) paper-based humidity sensors with polymer sensing elements.

Sensing Material/Paper	Method/Electrode	Range RH (%)	Response/Sensitivity	Res./Rec. Time	Ref.
PANI (photographic paper)	Inkjet printing/	13–95	~2.5 (R_0_/R); 220 Ω/% RH (ΔR/% RH)	N/A	[77]
PANI-CMC copolymer (glass)	Spin coating/-	5–95	~2.5 (R_0_/R); 2.5 MΩ/% RH (ΔR/% RH)	45/60 s	[78]
PANI/CNF sheet (cellulose sheet)	-/-	30–50	1.3 µA/% RH (ΔI/% RH)	370/1500 s	[79]
PANI/SLS (highly porous cellulose paper)	Polymerization/ Ag, printing	5–95	~120 (R_0_/R); 35 kΩ/% RH 99.2% (ΔR/R)	18/35 s	[80]
PANI/RGO (polypropylene filter paper)	Filtration/Ag	11–98	580% (ΔI/I_0_)	50/100 s	[81]
Ng PANI/paper composite (filter paper)	Polymerization/ Copper tape	20–95	~10 (R_0_/R); 10 kΩ/% RH	1300/2800 s	[82]
Nf PANI/paper composite		75–95	~5 (R/R_0_); 50 Ω/% RH	N/A	
PEDOT: PVMA (photographic paper)	Inkjet printing/-	11–98	71–98% (ΔR/R); 3–50 (R_0_/R)	<5/<5 s	[83]
Polypyrrole (chromatography paper 1CHR)	Polymerization/ graphite, printing	33–90	~1.3 (R/R_0_); ~17 Ω/% RH (ΔR/% RH)	N/A	[84]

Ng—nanogranular; Nf—nanofibrous; CMC—microcrystalline celluloșe; CNF—cellulose nanofibers; PANI—polyaniline; PDMS—polydimethylsiloxane; PEDOT—poly(3,4-Ethylenedioxythiophene); PVMA—copolymer P(VM-alt-MA); RGO—reduced; SLS—sodium lauryl sulfate.

**Table 7:** Summary of PB impedance type humidity sensors (f_0_ = 1 kHz).

Sensing Material	Method/Electrode	Range (% RH)	Response, (Z_0_/Z)	Res./Rec. Time	Ref
PEDOT:PSS (bond paper)	Inkjet printing	16–90	~3 (Z_0_/Z)	N/A	[89]
PANI (bond paper)	Inkjet printing	16–45 45–90	~10^4^ (Z_0_/Z) ~10 (Z_0_/Z)	9 min 12 min	
PANI (bond and photographic paper)	Inkjet printing	13–90	~2.5 (Z_0_/Z) −0.23 kΩ/% RH	N/A	[77]
PANI (chromatography paper)	Drop coating	0–97	48%	220/150 s	[94]
PANI-CMC composite (glass plate)	Spin coating	25–75 75–95	~3 × 10^2^ (Z_0_/Z) ~2 (Z_0_/Z)	10/90 s	[95]
Paper (print paper)	Pencil-trace electrodes	11–50 50–95	~3 (Z_0_/Z) 400 (Z_0_/Z)	N/A 56/86 s	[96]
PILs (print paper)	Drop casting	11–95	~10^3^ (Z_0_/Z)	13/145 s	
Paper (Whatman paper)	/PEDOT:PSS electrode, screen printing	25–60 60–85	<2 (Z_0_/Z) ~300 (Z_0_/Z)	N/A	[97]
Paper functionalized with NaCl	Spray coating, dip coating	25–60 60–85	<2 (Z_0_/Z) 2×10^3^ (Z_0_/Z)	N/A	
Paper (printing paper)	/Ag, screen printing	11–60 60–95	~2 (Z_0_/Z) ~65 (Z_0_/Z)	N/A 101/111 s	[98]
Paper functionalized with EPTAC	Immersion	11–95	~2×10^3^ (Z_0_/Z)	25/188 s	
TEMPO-oxidized cellulose nanofibers (CNF)	Free-standing film/PEDOT:PSS, Ag, screen printing	20–85	−0.1 Ω/% RH ~10^3^ (Z_0_/Z)	6–7/7–8 s	[99]
ZnO/CNF	Free-standing pellet/graphite pencil drawing	40–80	~5 (Z0/Z) −0.45 MΩ/% RH	N/A	[100]
		80–90	160 (Z0/Z) 4 MΩ/% RH		
CNF	Free-standing film/carbon, screen printing	20–90	47 (Z_0_/Z)	200/1020 s	[101]
CNF/PEG			1036 (Z_0_/Z)	265/490 s	

**Table 8:** Humidity-sensing properties of blank paper and PILs@Paper sensors.

Sensor	PILs Content	Sensitivity	Hysteresis (% RH)	Response/Recovery Times
Blank Paper	0%	408.2	19	56 s/86 s
PILs@Paper1	6.24%	408.4	11	45 s/99 s
PILs@Paper2	9.05%	616.5	9	38 s/105 s
PILs@Paper3	15.07%	961.3	7	25 s/113 s
PILs@Paper4	24.08%	1426.6	22	13 s/145 s
PILs@Paper5	34.02%	2662.4	25	7 s/202 s

Source: Reprinted with permission from [98]. Copyright: 2021: Elsevier.

**Table 9:** Performances of mass-sensitive PB humidity sensors.

Type	Sensing Material	Method	RH Range, %	Resonance Frequency	Frequency Shift	Res./Rec. Times	Ref.
QCM	Bacterial cellulose (BC)	Drop coating	5–55 55–97	5 MHz	7 Hz/% RH 43 Hz/% RH	N/A	[112]
	CNC	Air-brush method	11–60 60–97	10 MHz	60 Hz/% RH ~200 Hz/% RH	60/15 s	[106]
	Polydopamine/CNC/GO	Drop coating	10–60 60–97	10 MHz	26 Hz/% RH 90 Hz/% RH	11/4 s 37/5 s	[109]
	CNC	Drop coating	11–50 50–95	~5 MHz	~6 Hz/% RH ~40 Hz/% RH	90–120/50–60 s	[107]
	Nitro-modified CNC	Drop coating	11–84	20 MHz	28 Hz/% RH	18/10 s	[108]
SAW	BC	Spin coating	5–25 30–85 85–95	200 MHz	790 Hz/% RH ~1300 Hz/% RH ~3000 Hz/% RH	3/3 s 7/4 s 12/5 s	[110]
CMUT	CNC	Spin coating	11–53 53–94	10 MHz	900 Hz/% RH 2000 Hz/% RH	7/2 s	[111]

**Table 10:** Performances of fiber-optic PB humidity sensors that have been reported in the literature.

Sensing Material	Film Deposition Technique	RH Range, %	Method	Response	Res./Rec. Times	Ref.
HEC/PVDF	Drop coating	50–80	Light intensity	0.0228 dB/% RH	N/A	[115]
HEC/PVDF	Drop coating	50–80	Light intensity	0.03 dB/% RH	N/A	[114]
Carboxymethyl cellulose (CMC)	Deep coating	20–70 70–85	Light intensity	0.07 dB/% RH 0.86 dB/% RH	N/A	[116]
CMC	Deep coating	35–85	Wavelength change	0.17 nm/% RH	3/3 s	[118]
CMC/CNTs				0.23 nm/% RH		
CLC	Program-controlled coating system	38–98	Wavelength change	0.28 nm/% RH	30 min/	[117]
Cellulose-acetate butyrate (CAB)	N/A	10–95	Wavelength change	~0.5 nm/% RH	125/ s	[119]
CMC fiber	Dry-jet wet spinning	50–90	Light intensity	N/A	N/A	[120]

**Table 11:** Performances of microwave-fiber PB humidity sensors.

Sensing Material	Method	RH Range, %	Resonance Frequency	Response	Res./Rec. Times	Ref.
TEMPO-oxidized CNF	Films and sheet	5–65 65–97	f_0_ = 4.7 GHz	~0.3 MHz/% RH ~3.6 MHz/% RH	N/A	[121]
		22–89	f_0_~8.1 GHz	2.5 MHz/% RH	N/A	[122]
TEMPO-oxidized CNF/polyvinyl alcohol (PVOH)		22–89	f_0_~6.9 GHz	~4 MHz/% RH	N/A	
PEDOT:PSS/CNF		0–90	f_0_ = 2.78 GHz	N/A	N/A	[123]

**Table 12:** A comparison between different paper-based RFID humidity sensors.

Ref.	*f*_0_	Dimension	Sensitivity	Quality Factor
[137]	24 MHz	-	13 kHz/% RH	-
[133]	157 MHz	402 mm^2^	370 kHz/% RH	3.7
[136]	182 MHz	202 mm^2^	140 kHz/% RH	6.5

**Table 13:** Humidity indicators based on cellulose nanocrystals.

Sensing Material	RH Range. %	λ_max_ (Reflection) Change, nm	Response/Recovery Times	Ref.
CNC	70–95	500–550	7 min/>5 min	[167]
CNC	43–98	360–525	N/A	[168]
CNC/poly(ethylene glycol) (PEG)	50–90	520–670	N/A	[169]
CNC/PEG	30–100	500–910	N/A	[170]
CNC/PEG/PBD-PEGE	30–100	558–805	N/A	[171]
CNC/glycerol	16–98	620–710	N/A	[172]
CNC/glycerol	33–98	525–820	/300 s	[173]
CNC/polyol	30–95	425–652 455–695 501–750	N/A	[174]
CNC/polyacrylamide (PAM)	11–97	590–708	2–3 min/	[175]
CNC/PNIPAM	70–90	424–518 524–654 600–745	30 s/180 s	[176]
CNC/PNIPAM	9–98	490–680	N/A	[177]
CNC/NaCl	50–95	520–680	240 min/30 min	[178]
CNC/NMMO	13–97	590–680	<2 min/~10 min	[179]
Au/CNC	35–70	N/A	/2 min	[180]
Au/(CHI/CMC-N_3_)_25_/Au	10–98	N/A	N/A	[181]

## Data Availability

Not applicable.
